# Integrated transcriptomic and metabolomic analyses elucidate the mechanism of flavonoid biosynthesis in the regulation of mulberry seed germination under salt stress

**DOI:** 10.1186/s12870-024-04804-3

**Published:** 2024-02-21

**Authors:** Yi Wang, Wei Jiang, Chenlei Li, Zhenjiang Wang, Can Lu, Junsen Cheng, Shanglin Wei, Jiasong Yang, Qiang Yang

**Affiliations:** 1 College of Horticulture and Landscape Architecture, Zhongkai University of Agriculture and Engineering, Guangzhou, 510225 Guangdong China; 2 Sericultural & Agri-Food Research Institute Guangdong Academy of Agricultural Sciences, Guangzhou, 510610 China

**Keywords:** Mulberry, Salt stress, Seed germination, Flavonoids, Metabolomics, Transcriptome

## Abstract

Seed propagation is the main method of mulberry expansion in China, an important economic forest species. However, seed germination is the most sensitive stage to various abiotic stresses, especially salinity stress. To reveal the molecular regulatory mechanism of mulberry seed germination under salt stress, flavonoid metabolomics and transcriptomics analyses were performed on mulberry seeds germinated under 50 and 100 mmol/L NaCl stress. Analysis of the flavonoid metabolome revealed that a total of 145 differential flavonoid metabolites (DFMs) were classified into 9 groups, 40 flavonols, 32 flavones, 16 chalcones and 14 flavanones. Among them, 61.4% (89) of the DFMs accumulated continuously with increasing salt concentration, reaching the highest level at a 100 mmol/L salt concentration; these DFMs included quercetin-3-O-glucoside (isoquercitrin), kaempferol (3,5,7,4'-tetrahydroxyflavone), quercetin-7-O-glucoside, taxifolin (dihydroquercetin) and apigenin (4',5,7-trihydroxyflavone), indicating that these flavonoids may be key metabolites involved in the response to salt stress. Transcriptional analysis identified a total of 3055 differentially expressed genes (DEGs), most of which were enriched in flavonoid biosynthesis (ko00941), phenylpropanoid biosynthesis (ko00940) and biosynthesis of secondary metabolites (ko01110). Combined analysis of flavonoid metabolomic and transcriptomic data indicated that phenylalanine ammonia-lyase (*PAL*), 4-coumarate-CoA ligase (*4CL*), chalcone synthase (*CHS*), flavonol synthase (*FLS*), bifunctional dihydroflavonol 4-reductase/flavanone 4-reductase (*DFR*) and anthocyanidin reductase (*ANR*) were the key genes involved in flavonoid accumulation during mulberry seed germination under 50 and 100 mmol/L NaCl stress. In addition, three transcription factors, MYB, bHLH and NAC, were involved in the regulation of flavonoid accumulation under salt stress. The results of quantitative real-time PCR (qRT‒PCR) validation showed that the expression levels of 11 DEGs, including 7 genes involved in flavonoid biosynthesis, under different salt concentrations were consistent with the transcriptomic data, and parallel reaction monitoring (PRM) results showed that the expression levels of 6 key enzymes (proteins) involved in flavonoid synthesis were consistent with the accumulation of flavonoids. This study provides a new perspective for investigating the regulatory role of flavonoid biosynthesis in the regulation of mulberry seed germination under salt stress at different concentrations.

**Supplementary Information:**

The online version contains supplementary material available at 10.1186/s12870-024-04804-3.

## Introduction

Soil salinity is one of the major abiotic ecological stressors for horticultural crops and can cause ion toxicity, osmotic stress and nutrient deficiencies in plants [1, 2]. Approximately 20% of arable land worldwide is threatened by soil salinity, which severely affects plant growth and development and biomass accumulation and may lead to morphological, physiological, biochemical and molecular changes in crops [3, 4]. One of the important salts in soil is sodium chloride, which is highly soluble and ubiquitously distributed, and the impact of this salt on plant development and productivity has led to severe agricultural yield loss [5, 6]. Therefore, studying the effects of salt stress on plants and exploring the mechanisms of plant salt tolerance are of great significance for discovering salt tolerance-related genes, developing and utilising salt-tolerant plant resources, and improving plant salt tolerance [7].

Mulberry (*Morus atropurpurea*) belongs to the Moraceae family and is considered the sole crop of economic importance in sericulture [8], and its various parts (leaves, stems and roots) are used in the preparation of various products for the pharmaceutical, food, cosmetic and health care industries [9, 10]. As an important economic forest species, mulberry is able to adapt to adverse abiotic stress conditions, including salinity, flooding, drought and metal stress, making it an excellent material for woody plant stress tolerance studies [11]. However, seed propagation is the main method of mulberry expansion. Seed germination is the most sensitive stage to various abiotic stresses, especially salinity stress. Although mulberry plants are highly promising ecological tree species for afforestation, soil and water conservation, bioremediation of pollutants, land desertification and saline land management [12, 13], the seed germination stage is the most sensitive period to abiotic stresses, especially salinity stress [14, 15]. In our previous research, we found that the expression levels of GDSL esterase/lipase, GDSL esterase/lipase 6, aspartic proteinase nepenthesin-2 and bark storage protein A were significantly upregulated during mulberry seed germination under salt stress, indicating that these proteins may promote the tolerance of mulberry seed germination to salt stress [15]. Furthermore, the response of mulberry to salt stress is influenced by multiple factors and involves the expression and regulation of many resistance genes; for example, overexpression of the mulberry *RACK1* gene in *A. thaliana* reduced tolerance to drought and salt stress, and the expression level of the gene was altered in response to stress and stimuli [16]. The *MuGABA-T* gene is constitutively expressed at different levels in mulberry tissues, and its expression is substantially induced by NaCl, abscisic acid (ABA) and salicylic acid (SA) [17].

Mulberry plants are rich in flavonoids and other secondary metabolites, such as phenylpropanoids and alkaloids, which have a wide range of effects on the defense against biotic and abiotic stresses [18]. Flavonoids are an important family of secondary metabolites involved in plant development and defense and have various functions; for example, they can act as antioxidants, antibacterial agents and free-radical scavengers [19]. Based on the draft sequence of the mulberry genome, most of the genes associated with the biosynthesis of the flavonoid skeleton in *Morus notabilis* have been identified [20], including those encoding *PAL*, cinnamate 4-hydroxylase (*C4H*), *4CL*, *CHS*, chalcone isomerase (*CHI*), flavanone 4-reductase (*FNR*), flavone 3-hydroxylase (*F3H*) and *FLS* [21]. In addition, studies have shown that flavonoid accumulation in plants enhances tolerance to abiotic stresses [22]. The expression of *EaCHS1* and *AeCHS* in transgenic *Eupatorium adenophorum* and *Abelmoschus esculentus* plants, respectively, regulated flavonoid accumulation and improved resistance to salt stress during seed germination and root development by maintaining reactive oxygen species (ROS) homeostasis [23, 24]. Overexpression of the *CrUGT87A1* gene in *Carex rigescens* in *Arabidopsis thaliana* enhanced plant salt tolerance by increasing the flavonoid content [25]. Overexpression of the *AtMYB12* gene in *A. thaliana* significantly increased flavonoid accumulation and enhanced salt and drought tolerance in transgenic *A. thaliana* plants [26]. Salt stress induces phosphorylation of the GmMYB173 protein, which increases its affinity for the GmCHS5 promoter and promotes the transcription of GmCHS5, a process that may improve salt tolerance in soybean by promoting the accumulation of dihydroxy B-ring flavonoids [27]. These studies indicate that flavonoids play important regulatory roles in the physiological and molecular mechanisms involved in the regulation of salt tolerance in plants.

Seed germination is the first and most critical stage of plant morphogenesis, growth and development and is a key factor determining the quality of seedling growth [28]. The inhibition of seed germination by salt stress is mainly manifested by osmotic stress, the accumulation of excess ROS and the disruption of cell structure, which together reduce the germination rate and prolong the germination time [5]. Our previous work on salt stress in mulberry seeds revealed that salt stress inhibited the germination rate and length of mulberry plants, significantly increased the activities of superoxide dismutase, peroxidase and catalase [15]. Therefore, resolving the metabolic regulatory mechanisms of metabolites (especially flavonoid compounds) and genes involved in the regulation of the salt-tolerant seed germination process is crucial for the breeding of salt-tolerant mulberry plants. Flavonoids are commercially important medicinal substances that can participate in plant seed germination under environmental stress [29]. According to a previous report, salt stress significantly inhibited the germination of leguminous crops (including *Lathyrus sativus*, *Vicia sativa*, and *Vigna unguiculata*) but increased the content of flavonoid compounds in their seeds [30]. Moreover, salt stress reduces the growth and development of *A. venetum* plants but increases the levels of quercetin and kaempferol [31]. Studies have also shown that appropriate salt treatment can effectively regulate the expression of structural genes in the flavonoid biosynthesis pathway, thereby affecting the production of flavonoids [32]. However, there is limited knowledge of the molecular regulatory mechanisms of flavonoid biosynthesis during mulberry seed germination under salt stress.

Therefore, in the present study, transcriptomic and metabolomic analyses were further utilized to explore the molecular regulatory mechanisms involved in mulberry seed germination under salt stress. In addition, we performed qRT‒PCR to verify the gene expression at the transcript level and employed PRM analysis to quantify the key enzymes involved in flavonoid biosynthesis. Our study elucidated the potential connection between key genes and metabolites involved in mulberry seed germination under salt stress and identified the types and variations in differentially accumulated flavonoid metabolites in mulberry seedlings with the objective of providing a scientific basis for enhancing salt tolerance traits.

## Materials and methods

### Seed germination and stress treatments

The experimental material of this study is *Morus atropurpurea* provided by the Institute of Sericulture and Agri-Food Research of Guangdong Academy of Agricultural Sciences. Mature mulberry seeds with full grains and a uniform shape and size were selected. The seeds were disinfected with 0.5% potassium permanganate for 3 min, washed with sterile water, and then soaked in distilled water for 24 h, after which filter paper was used to absorb water from the seed surface. One hundred seeds were placed in a Petri dish with 2 layers of filter paper and a diameter of 9 cm. Five milliliters of NaCl solution of different concentrations or distilled water was used to soak the filter paper, and the Petri dish was covered with a lid. Afterward, the filter paper was changed once a day and supplemented with NaCl solution and distilled water to ensure that the salt concentration in each dish remained constant. Three treatments were used in the germination test, namely, 0 (distilled water control), 50 and 100 mmol/L NaCl solutions (Ma0, Ma50 and Ma100, respectively), and each treatment was replicated three times. The Petri dishes were placed in an artificial climate incubator at a light/darkness of 16/8 h/d, a light intensity of 480 lx, a constant temperature of 25 °C and a relative humidity of 65%-75%. Germination was considered to occur when the radicle protruded through the seed coat. The number of germinated seeds was recorded daily. After 15 days of continuous incubation, the seedling samples were collected, the fresh weight of the whole plants was measured with an electronic analytical balance (3 replicates per treatment, 30 seedlings per replicate), and the root length was measured with a Vernier caliper (three biological replicates per treatment, 30 seedlings per replicate). The samples for morphological analysis were subsequently placed in a -80 °C freezer for metabolome and transcriptome analyses.

### RNA extraction, library construction and transcriptome sequencing

RNA isolation, purification and monitoring, cDNA library construction and RNA sequencing were performed as previously described [33]. In brief, to determine the purity, concentration and integrity of the RNA extracted in this study, a NanoPhotometer® spectrophotometer (IMPLEN, Westlake Village, CA, USA), a Qubit® RNA Assay Kit for the Qubit® 2.0 Fluorometer (Life Technologies, Carlsbad, CA, USA) and an RNA Nano 6000 Assay Kit for the Agilent Bioanalyzer 2100 system (Agilent Technologies, Santa Clara, CA, USA) were used for inspection, measurement and evaluation, respectively [34]. The NEBNext® Ultra™ RNA Library Prep Kit for Illumina® (NEB, Ipswich, MA, USA) was used to generate sequencing libraries, which were subsequently sequenced on an Illumina HiSeq platform [34].

High-quality clean data were obtained using sequencing by synthesis (SBS) technology. Clean reads were sequenced against the reference genome using HISAT2, and fragments per kilobase of transcript sequence per million mapped tags (FPKM) values were used for transcription level quantification. DESeq2 [35] was used to calculate the ratio of the FPKM (fold change) and false discovery rate (FDR) between the compared samples, using |log_2_Fold Change|≥ 1 and FDR < 0.05 as screening criteria to screen differentially expressed genes (DEGs). Gene Ontology (GO) enrichment and Kyoto Encyclopedia of Genes and Genomes (KEGG) pathway enrichment analyses of DEGs were performed using the R (v4.0.3) package clusterProfiler (v3.18.1).

### Metabolite extraction and analysis

Sample preparation, extraction analysis, metabolite identification and quantification were performed sequentially according to the standard procedures described by Chen et al. [36]. Seedlings from 15 days of seed germination under salt stress were vacuum freeze-dried and ground (30 Hz, 1.5 min) to powder form using a grinder (MM400, Retsch). Fifty milligrams of the sample powder was weighed, and 1200 μL of -20 °C precooled aqueous 70% methanol solution was added to the internal standard extract. The dissolved samples were refrigerated overnight at 4 °C, during which time they were vortexed 6 times to improve the extraction rate. After centrifugation at 12,000 r/min for 3 min, the supernatant was aspirated, and the samples were filtered through a microporous membrane (0.22 μm pore size) and stored in an injection vial for UPLC‒MS/MS analysis.

### UPLC conditions

The sample extracts were analyzed using a UPLC‒ESI‒MS/MS system (UPLC, ExionLC™ AD, https://sciex.com.cn/) and a tandem mass spectrometry system (https://sciex.com.cn/). Chromatographic separation was performed on an Agilent SB-C18 UPLC column (1.8 µm, 2.1 mm * 100 mm) using mobile phase A (pure water with 0.1% formic acid) and mobile phase B (acetonitrile with 0.1% formic acid). The elution gradient used was as follows: 95:5 v(A)/v(B) at 0 min, 5:95 v(A)/v(B) at 9 min, 5:95 v(A)/v(B) at 1 min, 95:5 v(A)/v(B) at 1.1 min, and 95:5 v(A)/v(B) at 2.9 min. The flow velocity was set to 0.35 mL/min, the column oven was set to 40 °C, and the injection volume was 2 μL.

The samples were separated by chromatography and then transferred to a mass spectrometry instrument, after which the effluent was alternately transferred to an electrospray ionization (ESI) triple quadrupole (QQQ) instrument. The mass spectrometry conditions were as follows: electrospray ion source temperature, 500 °C; ion spray voltage (IS), 5500 V (positive ion mode)/-4500 V (negative ion mode); ion source gas I (GSI) and gas II (GS II), 50 psi and 60 psi, respectively; curtain gas (CUR), 25 psi; and collision-activated dissociation (CAD) parameters, high. QQQ scans were acquired via multiple reaction monitoring (MRM) experiments with the collision gas (nitrogen) set to medium. In the triple quadrupole, each ion pair was scanned for detection according to the optimized declustering potential (DP) and collision energy (CE) [36].

### Qualitative and quantitative analysis of metabolites

The quantitative analysis of metabolites was performed using triple quadrupole mass spectrometry in MRM mode. The mass spectrometry data were processed using Analyst 1.6.3 software. Next, the detected metabolites were analyzed using unsupervised principal component analysis (PCA), and the analysis was performed with the prcomp function of R software (version 3.5.1) to obtain a preliminary idea of the overall metabolite differences between groups of samples and the magnitude of variability between samples within groups. Both hierarchical cluster analysis (HCA) and Pearson correlation coefficient (PCC) analysis were performed using the R package ComplexHeatmap (www.r-project.org). The differences between samples were then analyzed using orthogonal partial least squares discriminant analysis (OPLS-DA) to identify differentially abundant metabolites, and OPLS-DA model calculations were performed using the R package MetaboAnalystR (http://www. metaboanalyst.ca/). Variables with variable importance in projection (VIP) values greater than 1 were considered differential variables. A method combining the fold difference values and the VIP values of the OPLS-DA model was adopted to screen the differentially abundant metabolites with the criteria absolute log2-fold change (|log2FC|≥ 1.0) and VIP > 1. After normalization and centralized treatment, K-means analysis of differentially abundant metabolites was carried out to investigate the variation in relative metabolite content in different samples. Functional annotation of the differentially abundant metabolites was performed using the KEGG database (http://www.kegg.jp/kegg/compound/), and KEGG pathway enrichment analysis was performed based on the annotation results.

### Integrated analysis of the metabolome and transcriptome

When performing KEGG annotation, differentially abundant metabolites were annotated to multiple metabolic pathways simultaneously with DEGs, and gene pathways with a *p* value < 0.05 and metabolic pathways with a *p* value < 0.05 were selected to screen out the relevant metabolic pathways. For the joint analysis of the metabolome and transcriptome, log2 transformations were performed on the data. Screening was performed using the PCC and the corresponding p value with the criteria PCC > 0.80 and *p* value < 0.05. The correlations between metabolites and genes are represented by network diagrams, and the results for DEGs and differentially abundant metabolites with a PCC > 0.80 and a *p* value < 0.05 in each pathway were selected for graphing.

### Quantitative real-time PCR (qRT‒PCR) and PRM analysis

To verify the accuracy and reliability of the transcriptome sequencing results, 11 DEGs were selected for qRT‒PCR analysis. RNA was extracted using an OminiPlant RNA (Dnase I) (Cowin Biotech, Jiangsu, China) kit, and cDNA was synthesized using a MonScript™ kit (Monad Biotech, Wuhan, China) according to the manufacturer's instructions. Actin was used as a reference gene [37], and the QuantiNova SYBR Green PCR Kit (Qiagen, Shanghai, China) was used for qRT‒PCR detection. Each sample was tested three times. Relative expression levels were calculated according to the 2^−ΔΔCT^ method [38].

PRM analyses were performed according to Wang et al. [15]. Briefly, PRM was performed using Xcalibur software (Thermo Scientific, USA). An aliquot of approximately 1 µg of digested peptide was removed from each sample and mixed with an aliquot of 20 fmol of standard peptide (PRTC:ELGQSGVDTYLQTK) for detection. Analyses were performed on a Q Exactive Plus mass spectrometer equipped with an Easy nLC-1200 system (Thermo Fisher Scientific, Bremen, Germany). Two microliters of each sample was injected into the column at a flow rate of 300 nL/min for gradient separation, and the samples separated by HPLC were then analyzed by PRM for 60 min using a Q Exactive HF mass spectrometer (Thermo Scientific, USA). The analytical column was a homemade spiked column (75 µm × 200 mm, 3 µm-C18). Finally, the PRM data were statistically analyzed using Skyline software 3.5.0 (Thermo Scientific, USA).

### Statistical analysis of data

The data were precollated using Microsoft Excel 2010, and the results were visualized using SPSS software (version 22.0, SPSS, Inc., Chicago, IL, USA) and are expressed as the mean ± standard deviation (SD) of at least three measurements. One-way analysis of variance (ANOVA) and Duncan's method were used to analyze the significance of differences between samples, with the significance level set at 0.05.

## Results

### Morphological characteristics of mulberry seedlings under different salt concentrations

**Fig. 1:**
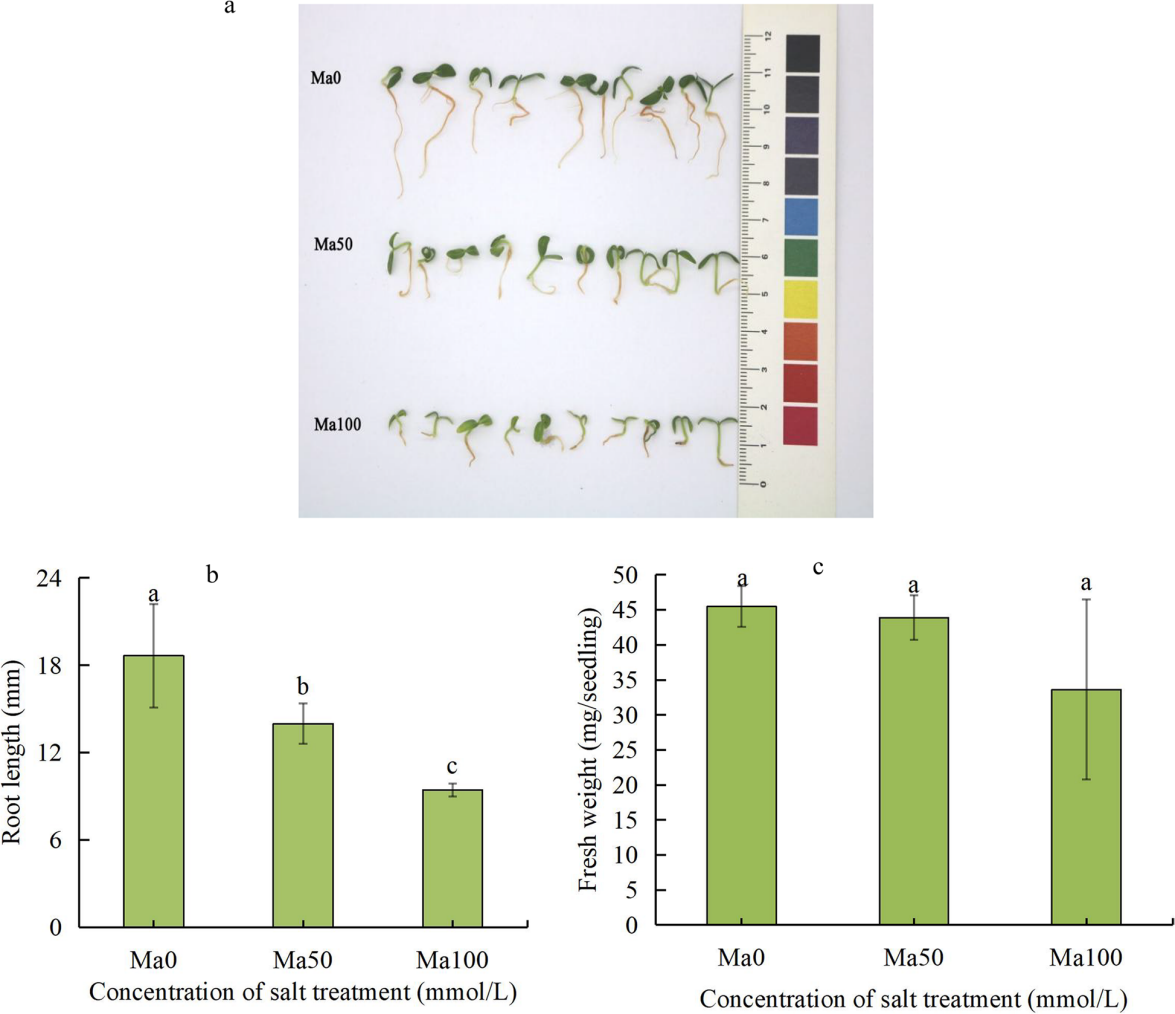
Effect of different salt treatment concentrations on mulberry seedling growth. **a** Phenotype of mulberry seedlings. **b** Root length (mm) of mulberry plants. **c** Fresh weight of mulberry plants. Ma0, Ma50 and Ma100 represent the 0 (distilled water control), 50 and 100 mmol/L NaCl treatments, respectively. At least three replicates were performed. The values are presented as the means ± SDs. Significant differences are indicated by different lowercase letters (*P* < 0.05)

After 15 days of salt stress treatment, the growth of the mulberry plants in the treatment group was slower than that in the control group. Root growth was obviously inhibited, and the roots were thin and yellowish and had few fibrous roots. After treatment with 100 mmol/L NaCl solution, the leaves of the mulberry plants turned yellow (Fig. 1a). Under salt stress, the length and fresh weight of the roots of the mulberry plants were significantly lower than those of the Ma0 plants (Fig. 1b, c). It was clear that salt stress inhibits the growth and development of mulberry plants.

### Transcriptome sequencing and functional signatures of differentially expressed genes

To investigate the effect of salt treatment on gene transcription, we sequenced and assembled the transcriptomes of 9 samples from the salt-treated and control groups. The RNA-seq dataset contained 59.91 Gb of clean data, and the clean data of each sample reached 5 Gb. A total of 39.68–47.58 million raw reads were obtained using the Illumina HiSeq platform. After removing reads containing adapters or poly-N and low-quality reads, high-quality data with Q30 percentages of 93.57%-94.28% and GC percentages of 46.89%-47.93% were obtained for analysis (Table S1). By alignment to the reference database, all the unigenes were successfully annotated to 35,579,703 (77.70%), 34,546,805 (77.96%), 33,183,223 (76.69%), 38,026,761 (81.07%), 37,280,630 (81.73%), 36,672,761 (81.59%), 35,364,413 (77.67%), 32,249,123 (73.31%) and 27,897,786 (71.53%). These results showed that the sequencing quality was sufficient for further analysis.

**Fig. 2:**
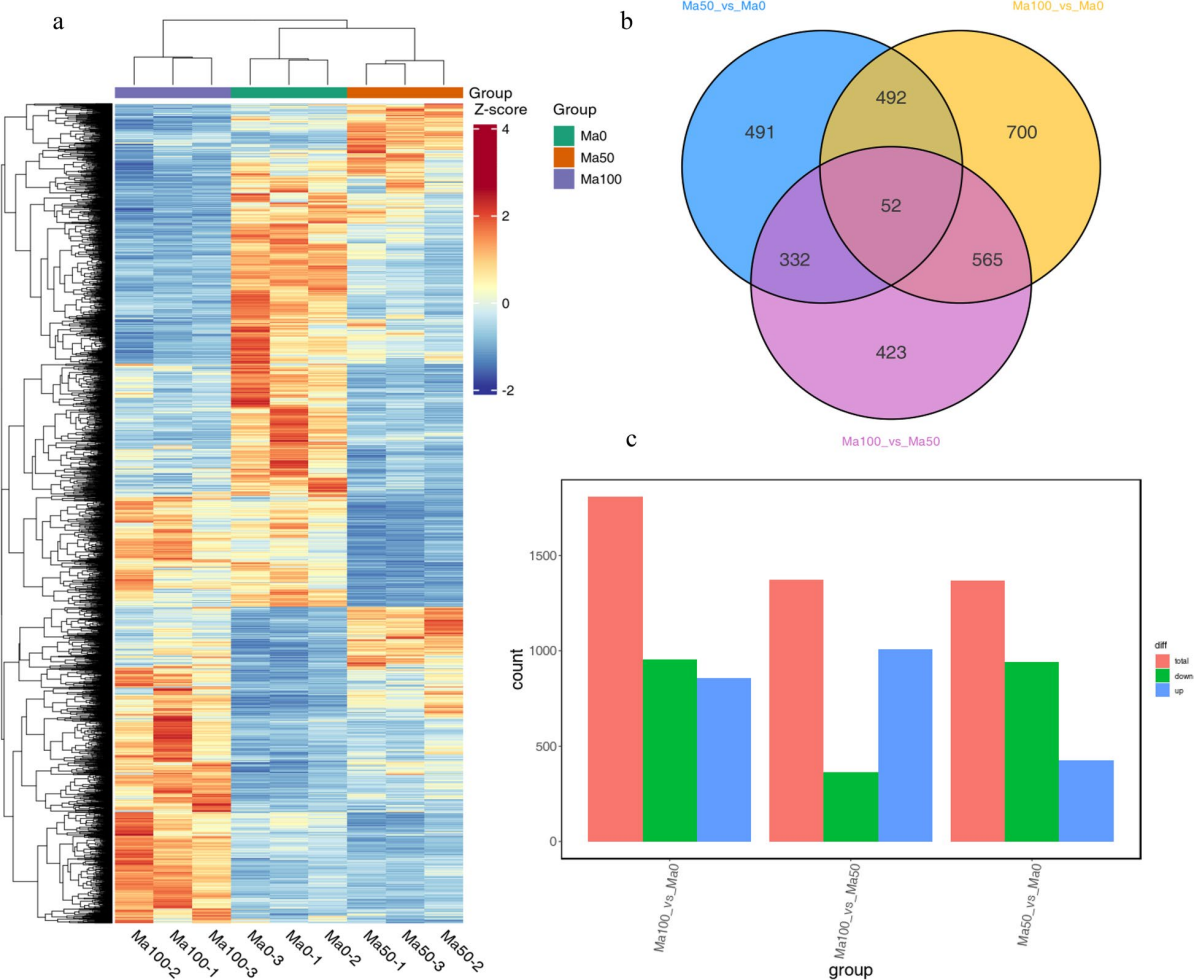
Differential expression analysis of Ma0, Ma50 and Ma100. **a** Expression of DEGs at different salt treatment concentrations. **b** Three-way Venn diagram showing the number of common DEGs and distinctive DEGs in different comparisons. **c** Numbers of DEGs in each comparison

To identify DEGs associated with mulberry flavonoid synthesis, FC ≥ 2 and FDR < 0.05 were used as screening criteria in this study. A total of 3055 DEGs were detected, and the expression of these DEGs in the Ma50 and Ma100 groups significantly differed from that in the Ma0 group (Fig. 2a). We identified 1367 DEGs (425 upregulated and 942 downregulated) by comparing the Ma50 and Ma0 treatments, including 4-coumarate-CoA ligase, chalcone synthase and putative anthocyanidin, ect; 1809 DEGs (856 upregulated and 953 downregulated) by comparing the Ma100 and Ma0 treatments, including chalcone synthase, bifunctional dihydroflavonol 4-reductase/flavanone 4-reductase and anthocyanidin reductase, etc.; and 1372 DEGs (1007 upregulated and 365 downregulated) by comparing the Ma100 and Ma50 treatments, including phenylalanine ammonia-lyase, transcription factor MYB14 and transcription factor bHLH92, etc. (Fig. 2b, c).

**Fig. 3:**
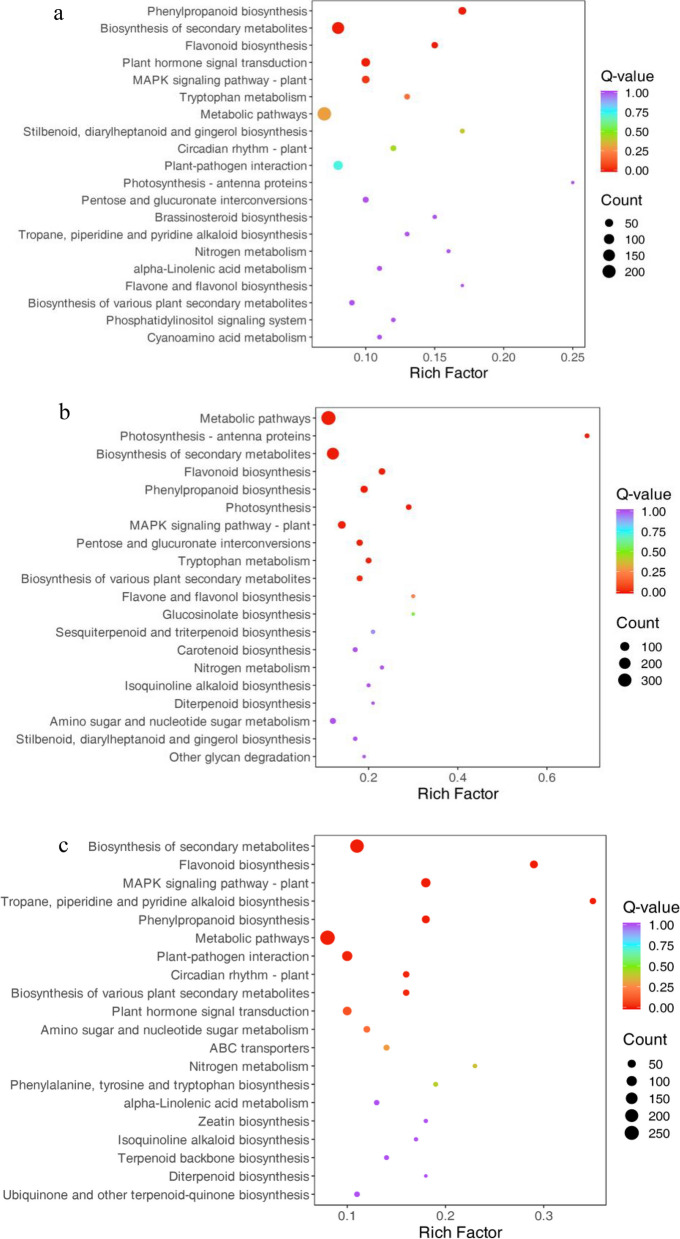
KEGG pathway enrichment of DEGs in the three comparison groups. Top 20 enriched KEGG pathways of DEGs in Ma50 vs. Ma0 **a** Ma100 vs. Ma0 **b** and Ma100 vs. Ma50 **c** The larger the Rich factor is, the greater the enrichment. The smaller the q value is, the more significant the enrichment

We performed GO and KEGG pathway functional enrichment analyses using the Blast GO and KEGG pathway databases, respectively. The DEGs were divided into three major categories, namely, biological process (BP), cellular component (CC) and molecular function (MF). In the Ma50 vs. Ma0, Ma100 vs. Ma0 and Ma100 vs. Ma50 comparisons, the GO classifications had 34, 39, and 34 subcategories, respectively. Based on the mapped homology, the DEGs were further classified into 28, 32, and 28 functional subcategories in these 3 comparison groups (Fig. S1a, b, c). The DEGs in the BP category were both mainly matched and classified as cellular process, metabolic process or response to stimulus. The most abundant GO terms in the CC category included both “cellular anatomical entity” and “protein-containing complex”. In the MF category, most of the DEGs were involved in binding, catalytic activity and transcription regulator activity. According to the KEGG pathway enrichment analysis, DEGs were mapped to 100, 110 and 94 KEGG pathways in Ma50 vs. Ma0, Ma100 vs. Ma0 and Ma100 vs. Ma50, respectively. According to the KEGG pathway database, the main enriched metabolic processes found in these three comparison groups were flavonoid biosynthesis (ko00941), phenylpropanoid biosynthesis (ko00940) and biosynthesis of secondary metabolites (ko01110) (Fig. 3a, b, c).

### Comprehensive analysis of flavonoid compounds

**Fig. 4:**
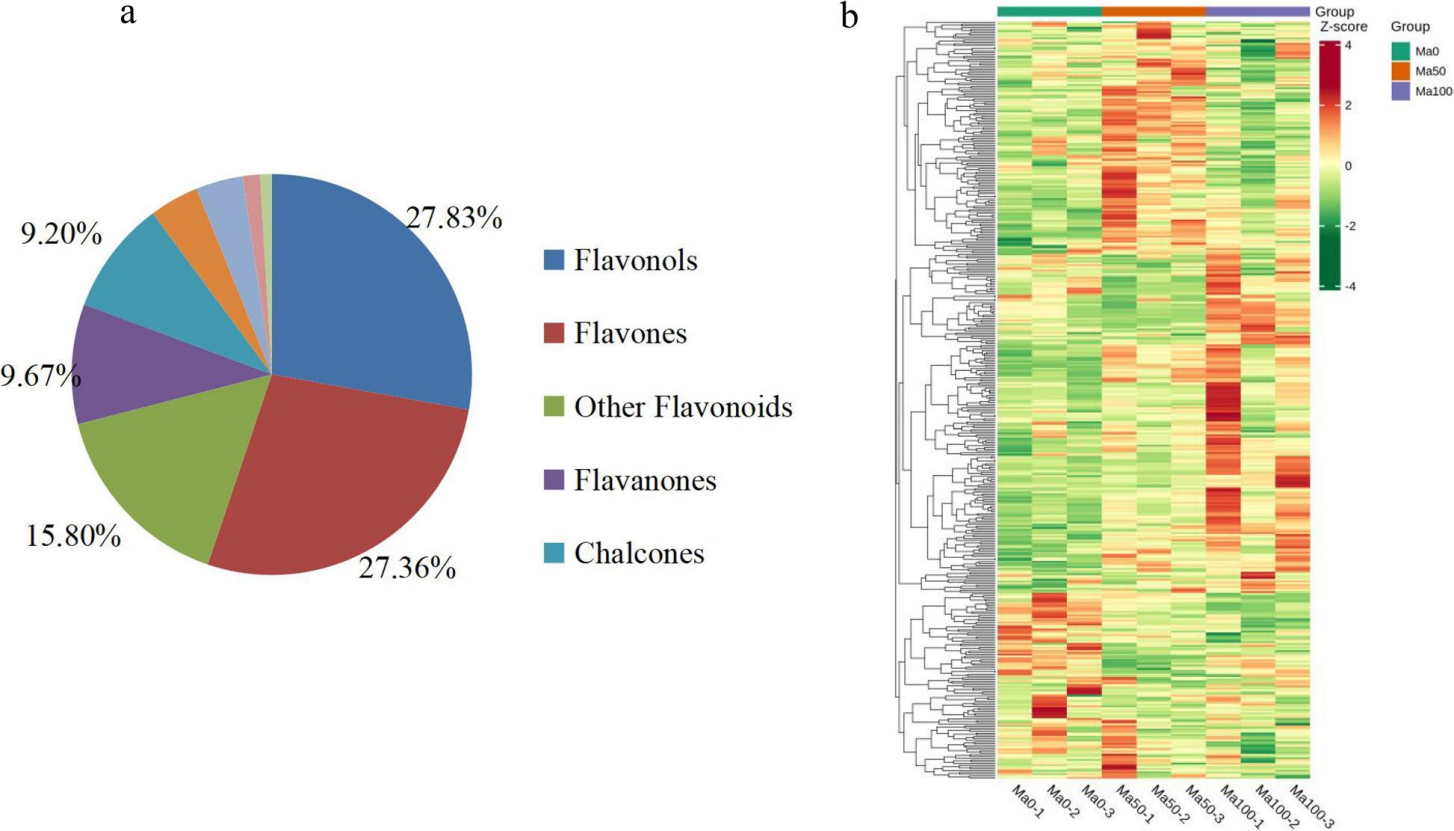
Data analysis of the identified flavonoids. **a** Composition analysis of the identified flavonoids. The five most common metabolites, flavonols, flavonoids, other flavonoids, flavanones and chalcones are shown next to the graph. **b** Heatmap of 424 flavonoid metabolites. The horizontal axis on the bottom shows the sample name, the vertical axis shows the metabolite information, the horizontal axis on the top indicates the grouping, and different colors indicate the different values obtained after standardization of the relative levels, where red represents high levels and green represents low levels

Widely targeted metabolite analysis via UPLC‒MS/MS was performed to analyze flavonoids in mulberry seedlings comprehensively after salt stress. The flavonoid metabolites in mulberry were qualitatively and quantitatively analyzed via triple quadrupole screening of ions and detection of the signal intensity characteristics of the ions according to the standard constructed MetWare Metabolite Database (MWDB) (MetWare, Wuhan, China). A total of 424 flavonoid metabolites were detected in mulberry seedlings under salt stress, including 118 (27.83%) flavonols, 116 (27.36%) flavones, 67 (15.80%) other flavonoids, 41 (9.67%) flavanones, 39 (9.20%) chalcones, 17 (4.01%) flavanonols, 16 (3.77%) flavanols, 6 (1.42%) tannins and 4 (0.94%) proanthocyanidins (Fig. 4a, Table S2). To visualize the differences in metabolites between different salt treatments in germinating mulberry seeds, a cluster heatmap analysis of the different types of flavonoids was performed, and the results are shown in Fig. 4b.

### Metabolome profiling

**Fig. 5:**
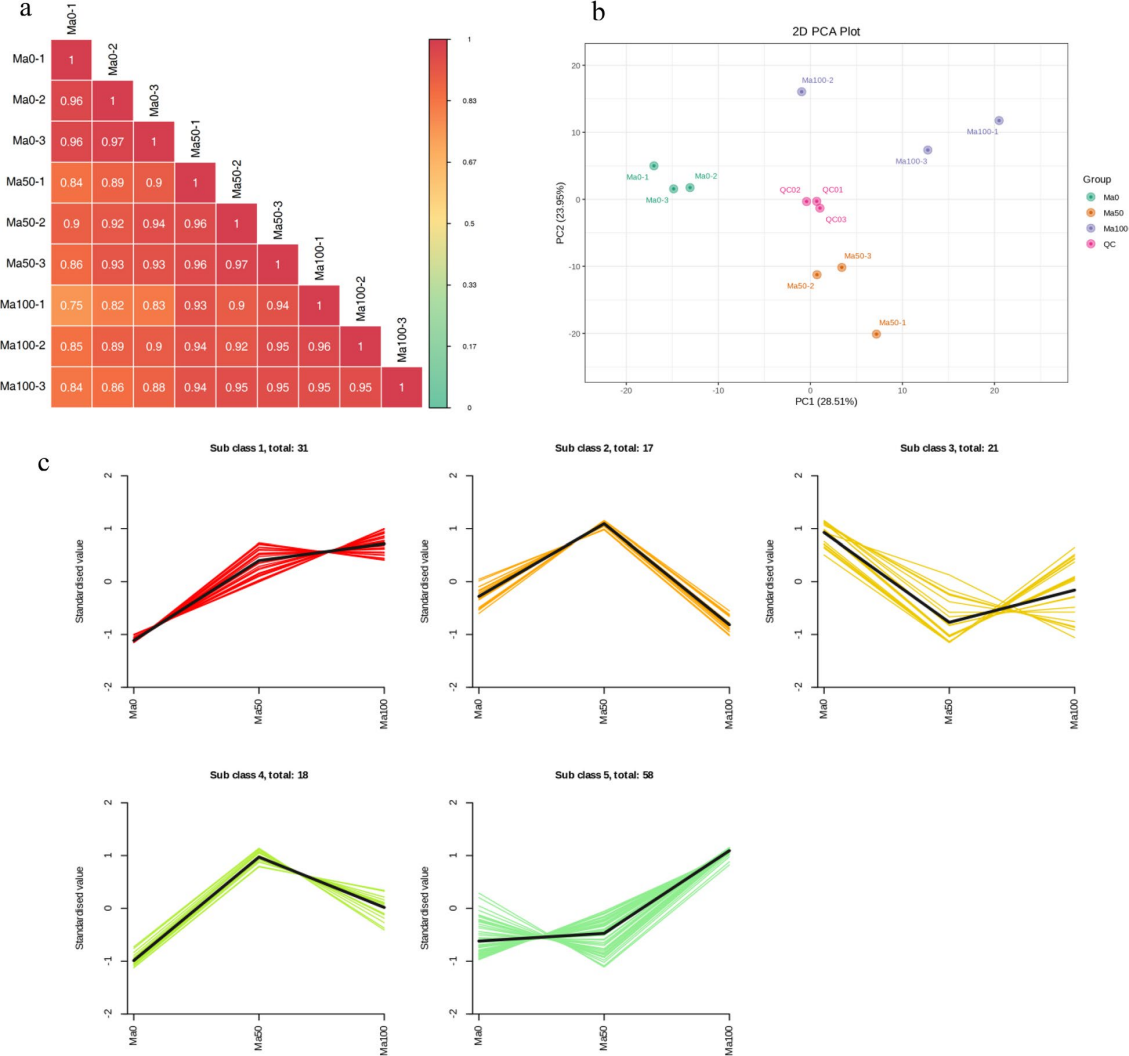
Quality control of metabolites identified in extracts of mulberry seedling samples. **a** Pearson correlation coefficients and **b** principal component analysis of metabolites extracted from control and treated mulberry plants after treatment with 0 mmol/L, 50 mmol/L and 100 mmol/L NaCl; each sample was analyzed in triplicate, and a quality control mixture was used for metabolomics. **c** K-means clustering analysis showing the dynamic accumulation of differentially abundant metabolites under different salt concentrations. The horizontal coordinate indicates the sample grouping, the vertical coordinate indicates the standardized metabolite relative content, ‘Subclass’ indicates the metabolite category number with the same change trend, and ‘total’ indicates the number of metabolites in that class. The data were plotted using the *prcomp* function with default settings in R software [39]

To better explore the potential pathways involved in salt stress tolerance, the seedling samples were grouped into 3 treatment groups of three biological replicates each for a series of qualitative and quantitative metabolite analyses. In this experiment, the PCC (R^2^) for biological replicate sequences was greater than 0.9 in the different comparison groups (Fig. 5a), and the correlation between replicate samples was strong. Subsequently, PCA was performed to determine the differences between treatment groups for quality control (QC) and to examine the magnitude of variability. The principal component scores showed that PC1 and PC2 explained 28.51% and 23.95%, respectively, of the variability between samples, with a total contribution of 52.46% (Fig. 5b). These sample groups showed a clear trend toward separation, demonstrating the reliability of the metabolomic data. K-means clustering was used to detect metabolites from the five clusters to examine changes in their relative levels in the sample group comparisons. Subclusters 1 and 5 contained 89 metabolites, and their levels in mulberry seedlings increased continuously with increasing salt concentration, peaking at a salt concentration of 100 mmol/L. These findings indicated that the flavonoid metabolites in subclusters 1 and 5 are closely related to salt tolerance and are key compounds involved in mulberry defense against salt stress. The representative compounds in these two subclusters included quercetin-3-O-glucoside (isoquercitrin), kaempferol (3,5,7,4'-tetrahydroxyflavone), quercetin-7-O-glucoside, taxifolin (dihydroquercetin) and apigenin (4',5,7-trihydroxyflavone). Subcluster 3 consisted of 21 metabolites whose levels decreased under the 50 mmol/L salt treatment and then increased significantly under the 100 mmol/L salt stress treatment. Interestingly, subclusters 2 and 4 contained 35 metabolites whose levels significantly increased under 50 mmol/L salt stress treatment and then decreased under 100 mmol/L salt stress treatment, suggesting that these metabolites may contribute to the regulation of salt stress in mulberry plants at low concentrations (Fig. 5c, Table S3).

**Fig. 6:**
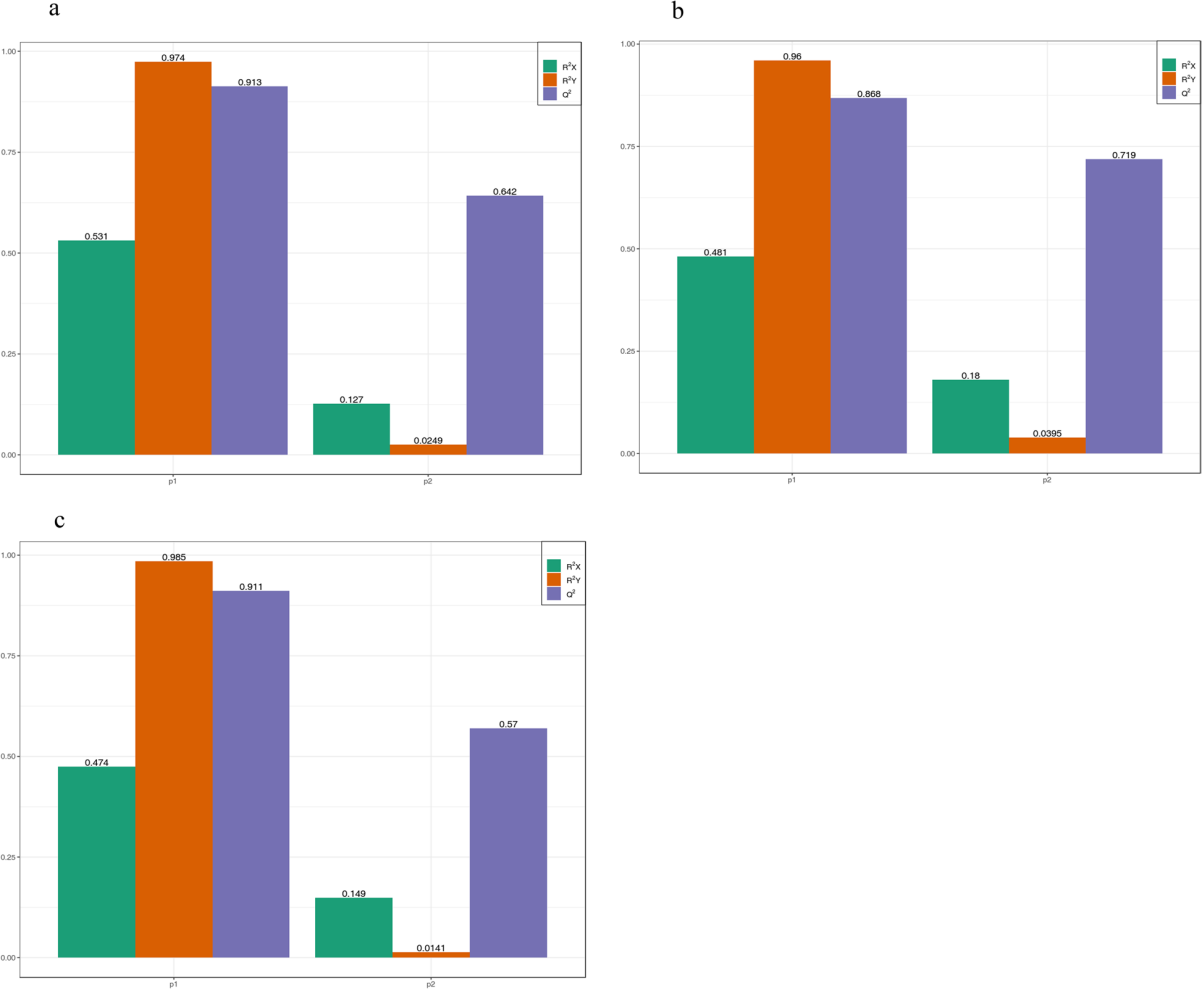
OPLS-DA model plots of the DFMs. **a** Ma50 vs. Ma0; **b** Ma100 vs. Ma0; **c** Ma100 vs. Ma50. P1 and P2 represent the first and second principal components of the model, respectively

Furthermore, the differences in flavonoid metabolites (DFMs) were examined for changes between comparison groups according to the OPLS-DA model. In the comparison between the 50 mmol/L salt stress treatment (Ma50) and the control group (Ma0), the values obtained were R^2^X = 0.531, R^2^Y = 0.974, and Q^2^ = 0.913 (Fig. 6a); in the comparison between the 100 mmol/L salt stress treatment (Ma100) and the control group, the values obtained were R^2^X = 0.481, R^2^Y = 0.960, and Q^2^ = 0.868 (Fig. 6b); and in the comparison between Ma100 and Ma50, the values obtained were R^2^X = 0.474, R^2^Y = 0.985, and Q^2^ = 0.911 (Fig. 6c). The results indicate the high reliability and stability of the models utilized.

**Fig. 7:**
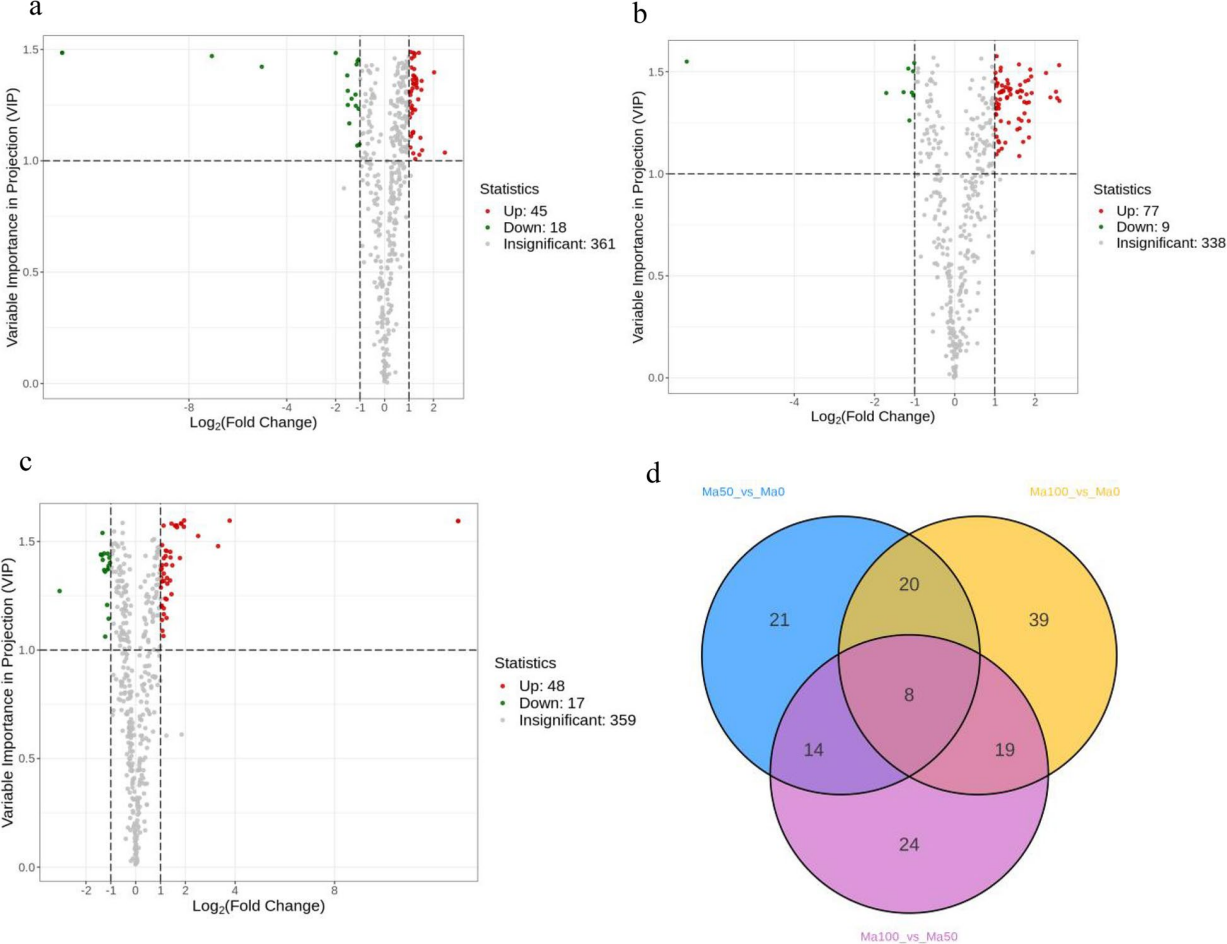
Volcano maps and venn diagram of the DFMs.** a** Volcano maps of Ma50 vs. Ma0; **b** Volcano maps of Ma100 vs. Ma0;** c** Volcano maps of Ma100 vs. Ma50; **d** Venn diagram showing the numbers of metabolites in Ma50 vs. Ma0, Ma100 vs. Ma0 and Ma100 vs. Ma50

Metabolites with a VIP value > 1, as well as a top fold change (FC) of ≥ 2 or ≤ 0.5, were taken as DFMs for discriminating the NaCl-treated group from the control group. The results of the screening of DFMs from the salt stress treatment and control groups are shown in Fig. 7. Overall, 63 DFMs (45 upregulated and 18 downregulated) were identified between Ma50 and Ma0 (Fig. 7a), 86 DFMs (77 upregulated and 9 downregulated) were identified between Ma100 and Ma0 (Fig. 7b), and 65 DFMs (48 upregulated and 17 downregulated) were identified between Ma100 and Ma50 (Fig. 7c, Table S4). Among these three comparison groups, the maximum number of DFMs was found in the 100 mmol/L NaCl treatment group, indicating that 100 mmol/L NaCl promoted the accumulation of flavonoids, thus contributing to the improvement of salt tolerance in mulberry plants (Fig. 7d). Moreover, 8 of these DFMs were found in the comparisons between Ma50 and Ma0, Ma100 and Ma0 and Ma100 and Ma50 (Fig. 7d).

**Fig. 8:**
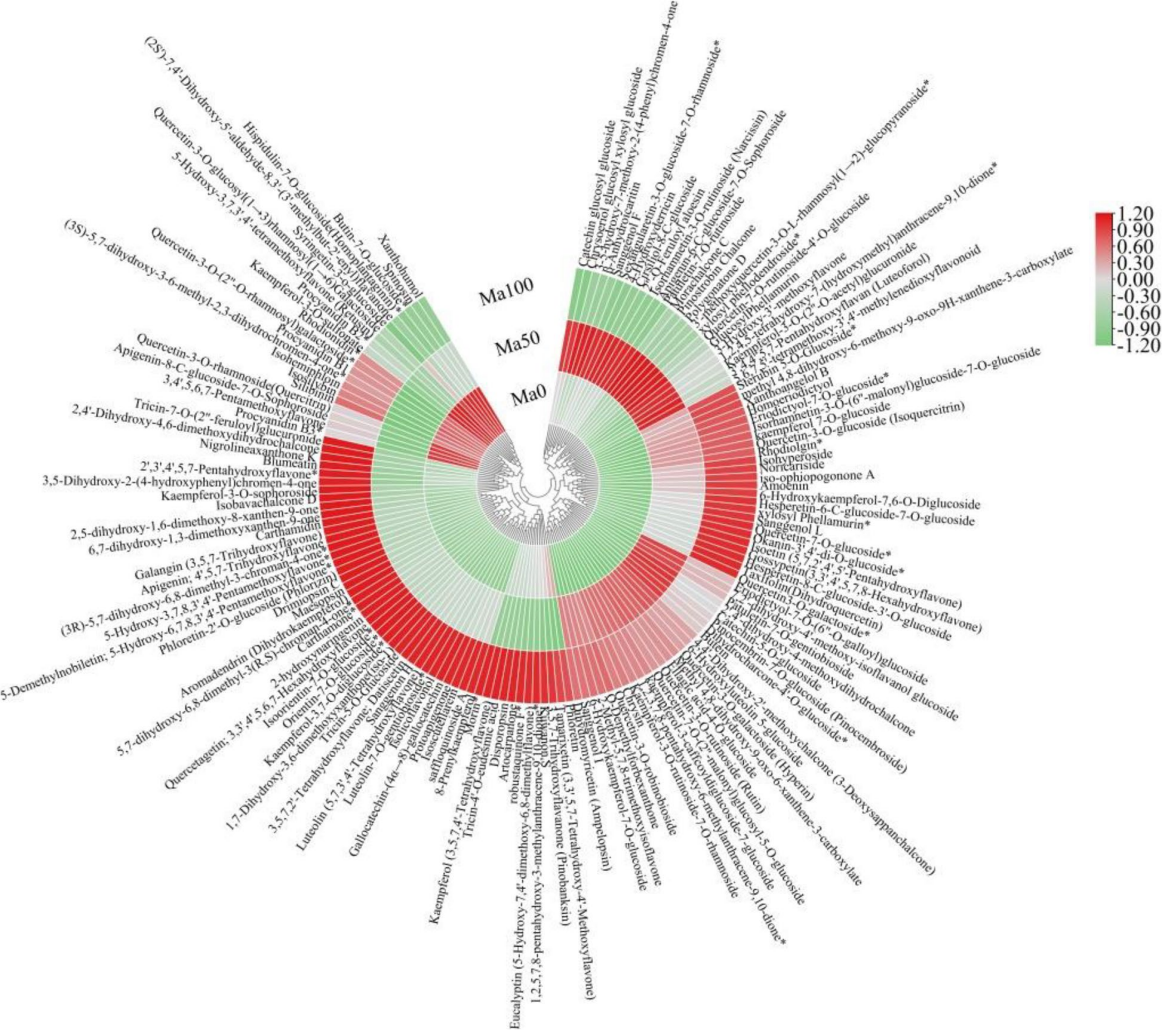
Heatmap of the changes in the DFM content among Ma0, Ma50 and Ma100. Red indicates elevated metabolite levels, and green indicates reduced metabolite levels

To further compare the patterns of changes in the levels of DFMs in mulberry seedlings under different salt treatments, we used TBtools to construct heatmaps of 145 DFMs from three sample groups of mulberry plants (Fig. 8). Elevated levels of metabolites are indicated in red, and reduced levels are indicated in green. Most of the DFM levels increased in Ma100, while a small number decreased. The DFMs that were significantly elevated in Ma100 and decreased in Ma0 and Ma50 included the following: among the 40 flavonols, the DFMs included quercetin-7-O-glucoside, kaempferol (3,5,7,4'-tetrahydroxyflavone), apigenin (4',5,7-trihydroxyflavone), quercetin-3-O-glucoside (isoquercitrin), amoenin, kaempferol-3,7-O-diglucoside, and 14 others; among the 32 flavones, the DFMs included orientin-7-O-glucoside, luteolin-7-O-gentiobioside, tricin-5-O-glucoside, chrysin, luteolin (5,7,3',4'-tetrahydroxyflavone), 2',3',4',5,7-pentahydroxyflavone, and 18 others; among the 28 other flavonoids, the DFMs included quercetin3-O-galactoside, sanggenon H, maesopsin, drimiopsin C, kuwanon S, and 12 others; among the 16 chalcones, the DFMs included phloretin-2'-O-glucoside (phlorizin), xanthoangelol B, carthamone, and 5 others; among the 14 flavanones, the DFMs included 2-hydroxynaringenin, sterubin 5-O-glucoside, eriodictyol-7-O-glucoside, and 7 others; and among the 8 flavanonols, the DFMs included aromadendrin (dihydrokaempferol), xylosyl phellamurin and taxifolin (dihydroquercetin). However, among the 3 flavanols, only gallocatechin-(4α → 8)-gallocatechin was significantly elevated in Ma100 and decreased in Ma0 and Ma50. Interestingly, among the 3 proanthocyanidins, procyanidin B1, procyanidin B2 and procyanidin B3 were significantly elevated in Ma0 and decreased in Ma50 and Ma100. Furthermore, 1-tannin (ellagic acid-4-O-glucoside) was elevated in Ma50 and Ma100 and decreased in Ma0.

**Fig. 9:**
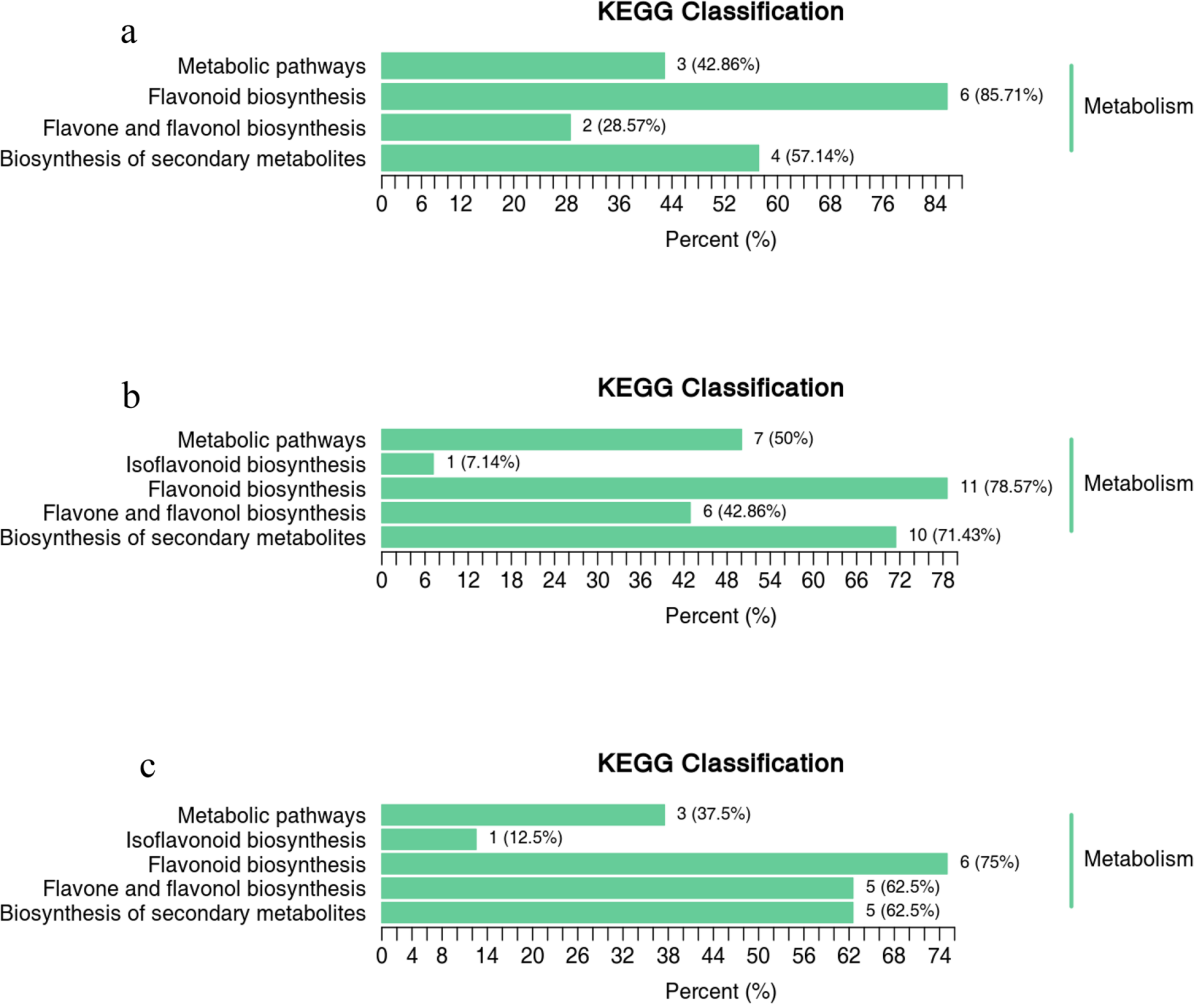
KEGG enrichment analysis of the differentially abundant metabolites in the comparisons of Ma50 vs. Ma0 **a** Ma100 vs. Ma0 **b** and Ma100 vs. Ma50 **c** The vertical coordinate represents the name of the KEGG metabolic pathway, and the horizontal coordinate represents the ratio of the number of differentially abundant metabolites annotated to that pathway to the total number of differentially abundant metabolites annotated

Different flavonoid metabolites can play unique roles in organisms, forming distinct metabolic pathways and biological pathways. A total of 63 flavonoid metabolites (including quercetin-3-O-rutinoside (rutin), kaempferol 7-O-glucoside, luteolin (5,7,3',4'-tetrahydroxyflavone) and catechin-5-O-glucoside, ect.) that were significantly differentially abundant between Ma50 and Ma0, 86 flavonoid metabolites (including quercetin-3-O-robinobioside, kaempferol (3,5,7,4'-tetrahydroxyflavone), dihydromyricetin (ampelopsin) and taxifolin (dihydroquercetin), ect.) that were significantly differentially abundant between Ma100 and Ma0, and 65 flavonoid metabolites (including quercetin-3-O-rhamnoside (quercitrin), kaempferol-3,7-O-diglucoside, apigenin; 4',5,7-trihydroxyflavone and morin, ect.) that were significantly differentially abundant between Ma100 and Ma50 in mulberry under salt stress were annotated using the KEGG database. The above annotated metabolites in each comparison group are shown in Fig. 9a, b, and c. KEGG enrichment analysis revealed that the significantly differentially abundant flavonoid metabolites were involved in pathways such as flavonoid biosynthesis (ko00941), secondary metabolite biosynthesis (ko01110), metabolic pathways (ko01100), and flavone and flavonol biosynthesis (ko00944).

### KEGG coenrichment analysis of differentially expressed genes and differentially abundant metabolites

**Fig. 10:**
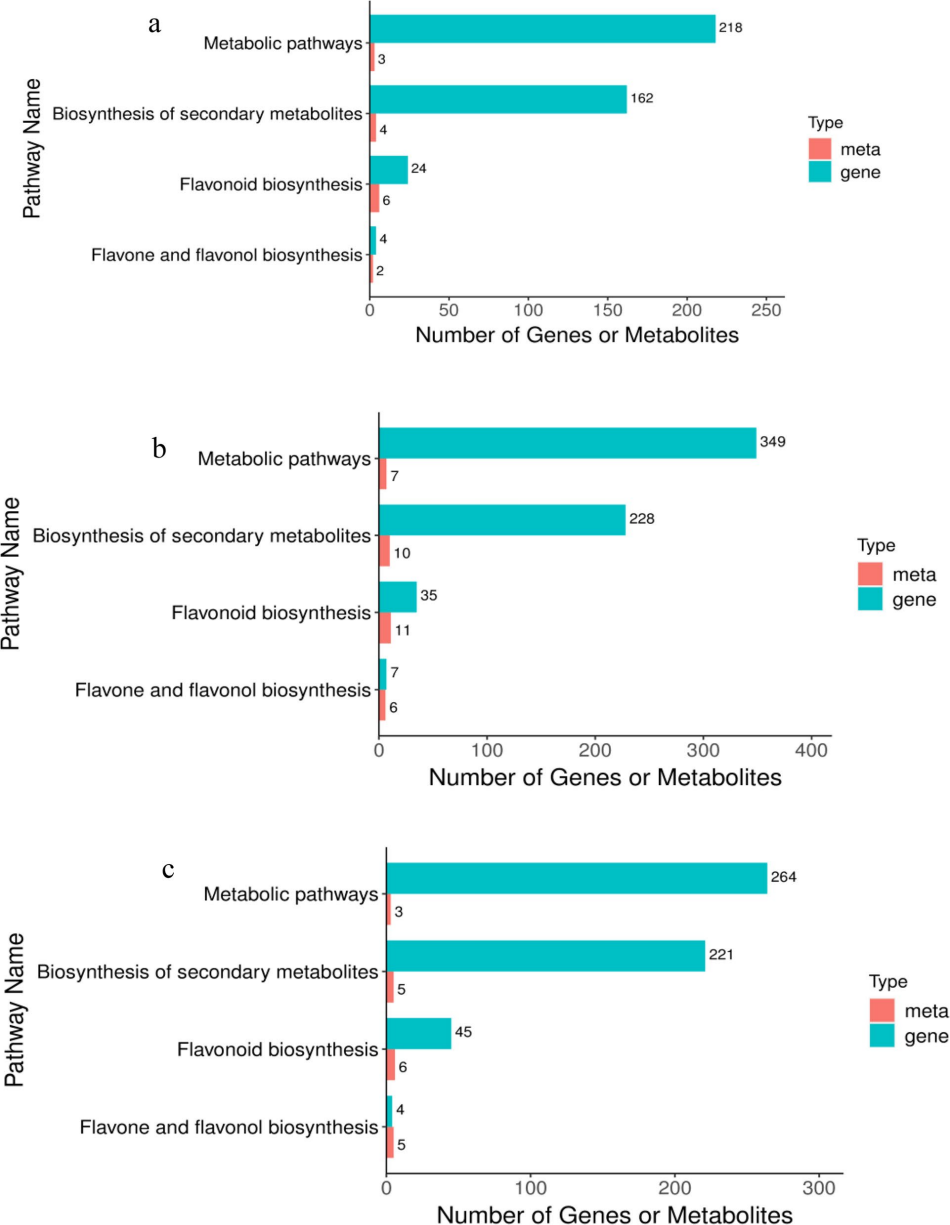
KEGG enrichment analysis bar graphs for Ma50 vs. Ma0 **a** Ma100 vs. Ma0 **b** and Ma100 vs. Ma50 **c** The horizontal coordinates represent the number of differentially abundant metabolites and DEGs enriched in the pathway, the vertical coordinates represent the KEGG pathway names, and the red and green bars represent the metabolome and transcriptome, respectively

Bar graphs were drawn using the KEGG pathways enriched in both histologies, and the bars show the number of differentially abundant metabolites and DEGs enriched in a particular pathway. If the number of shared KEGG pathways exceeded 25, only the top 25 pathways in terms of P value were shown based on the transcriptome, as shown in Fig. 10. KEGG analysis revealed four coenriched pathways, namely, metabolic pathways (ko01100), biosynthesis of secondary metabolites (ko01110), flavonoid biosynthesis (ko00941) and flavone and flavonol biosynthesis (ko00944), in the Ma50 vs. Ma0, Ma100 vs. Ma0 and Ma100 vs. Ma50 comparison groups. In the Ma50 vs. Ma0 comparison, these four coenriched pathways were significantly enriched in the transcriptome (*P* < 0.05) but not in the metabolome. In the Ma100 vs. Ma0 comparison, metabolic pathways were significantly enriched in both the transcriptome and metabolome, while the remaining three pathways were significantly enriched in both the transcriptome and the metabolome. In the Ma100 vs. Ma50 comparison group, metabolic pathways, biosynthesis of secondary metabolites, and flavonoid biosynthesis were significantly enriched in the transcription pathway but not in the metabolome. Flavone and flavonol biosynthesis were not significantly enriched in either the transcriptome or metabolome.

### Correlation network graph analysis of genes and metabolites

**Fig. 11:**
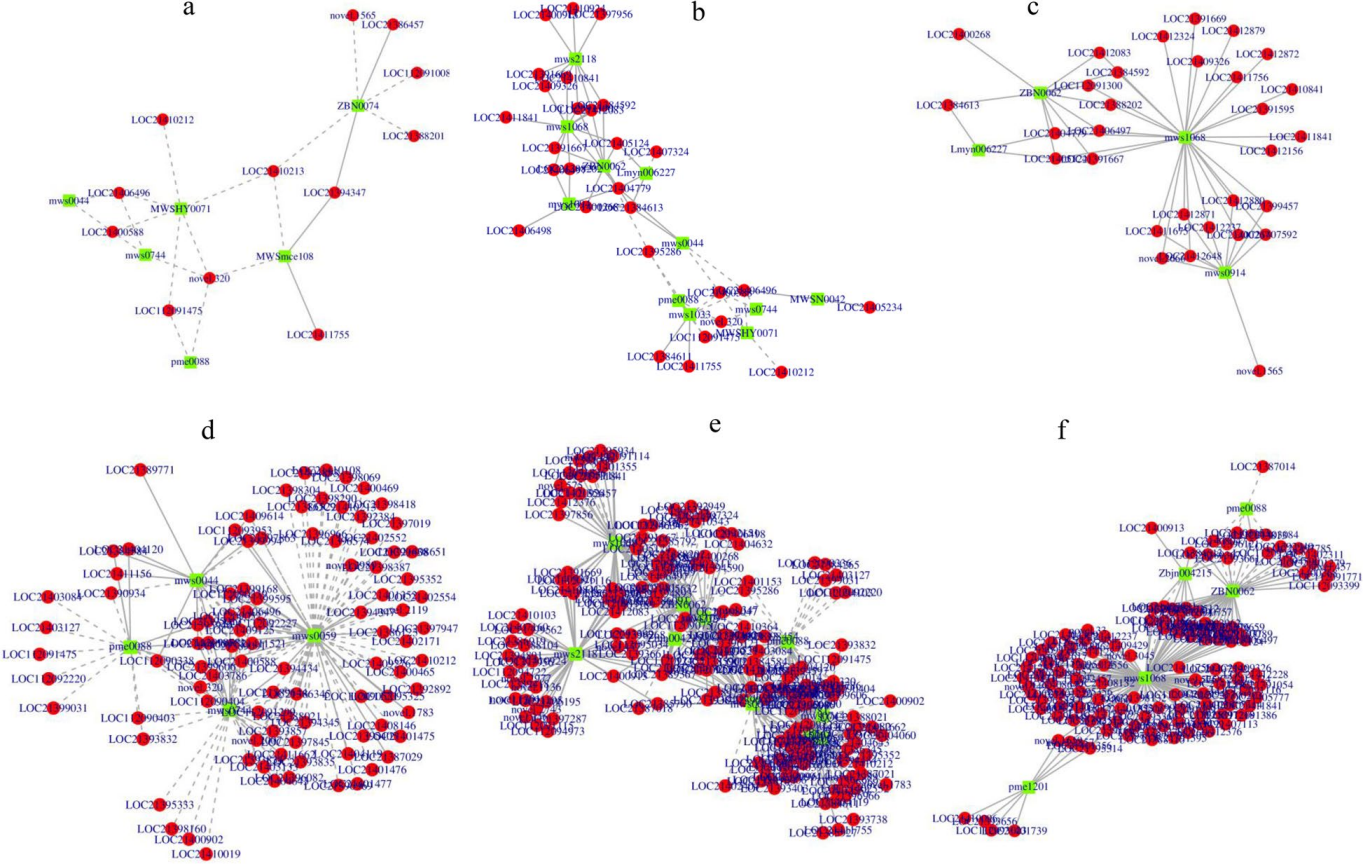
Gene and metabolite association analysis. For the ko00941 pathway, gene and metabolite association analysis was performed for Ma50 vs. Ma0 **a** Ma100 vs. Ma0 **b** and Ma100 vs. Ma50 **c** For the ko01110 pathway, gene and metabolite association analysis was performed for Ma50 vs. Ma0 **d** Ma100 vs. Ma0 **e** and Ma100 vs. Ma50 **f** Metabolites are marked with green squares, and genes are marked with red circles. Solid lines represent positive correlations, and dashed lines represent negative correlations

Subsequently, DEGs and DFMs involved in the flavonoid biosynthesis and biosynthesis of secondary metabolites were screened. Pearson correlation coefficient analysis of the genes and metabolites was performed to explore the regulatory mechanisms involved. In the flavonoid biosynthesis (ko00941) pathway, 12 genes were associated with 6 metabolites with differential abundance between Ma50 and Ma0, where butein (MWSmce108) was positively correlated with *CHS* (LOC21411755) and protein eceriferum 26-like (LOC21394347), dihydromyricetin (mws0744) was negatively correlated with *ANR* (LOC21400588), *FLS* (LOC21406496) and anthocyanin 5-aromatic acyltransferase (novel.320) (Fig. 11a). butein and dihydromyricetin were significantly upregulated in Ma50 and Ma0 (Table S4); Among the genes, 67 were associated with 11 metabolites with differential abundance between Ma100 and Ma0, where kaempferol (3,5,7,4'-tetrahydroxyflavone) (mws1068) was positively correlated with *CHS* (LOC112091300), *DFR* (LOC21384592); dihydrokaempferol (mws1094) and *DFR* ( LOC21404779), *FLS* (LOC21406497) and *FLS* (LOC21406498) were positively correlated; dihydroquercetin (mws0044) was negatively correlated with *ANR* (LOC21400588), *FLS* (LOC21406496); dihydromyricetin (mws0744) and *ANR* (LOC21400588), *FLS* (LOC21406496) were negatively correlated (Fig. 11b). kaempferol, dihydrokaempferol, dihydroquercetin and dihydromyricetin were all significantly upregulated in Ma100 and Ma0 (Table S4); Fifty genes were closely associated with 4 metabolites with differential abundance between Ma100 and Ma50, namely, kaempferol (3,5,7,4'-tetrahydroxyflavone) (mws1068), apigenin (4',5,7-trihydroxyflavone) (ZBN0062), 3,5,7-trihydroxyflavanone (pinobanksin) (mws0914) and galangin (3,5,7-trihydroxyflavone) (Lmyn006227). Among them, kaempferol (3,5,7,4'-tetrahydroxyflavone) was positively associated with 27 genes including *CHS* (LOC112091300), *DFR* (LOC21384592) and *FLS* (LOC21406497) (Fig. 11c). kaempferol was significantly upregulated in both Ma100 and Ma50 (Table S4). The results indicated that kaempferol, dihydrokaempferol, dihydroquercetin and dihydromyricetin may be involved as specific metabolites in the transcriptional regulation of key genes.

In the biosynthesis of secondary metabolites (ko01110) pathway, 161 genes were associated with 4 metabolites with differential abundance between Ma50 and Ma0, where quercetin-3-O-rutinoside (rutin) was positively correlated with phosphoenolpyruvate carboxykinase (ATP) (LOC21399168), pheophorbide a oxygenase (LOC21395013) and the UDP-glycosyltransferase 87A1 (LOC21394434) (Fig. 11d). A total of 440 genes were associated with 10 metabolites with differential abundance between Ma100 and Ma0, including kaempferol (3,5,7,4'-tetrahydroxyflavone) (mws1068), quercetin-3-O-glucoside (isoquercitrin) (mws0091), apigenin (4',5,7-trihydroxyflavone) (ZBN0062) and aromadendrin (dihydrokaempferol) (mws1094) (Fig. 11e). A total of 166 genes were associated with five metabolites with differential abundance between Ma100 and Ma50, including kaempferol (3,5,7,4'-tetrahydroxyflavone) (mws1068), phloretin (pme1201), apigenin (4',5,7-trihydroxyflavone) (ZBN0062), luteolin (5,7,3’,4’-tetrahydroxyflavone) (pme0088) and kaempferol-3-O-sophorosid (Zbjn004215), where kaempferol (3,5,7,4'-tetrahydroxyflavone) was positively correlated with 95 genes (Fig. 11f).

### Analysis of DEGs and DFMs associated with flavonoid biosynthetic pathways

**Fig. 12:**
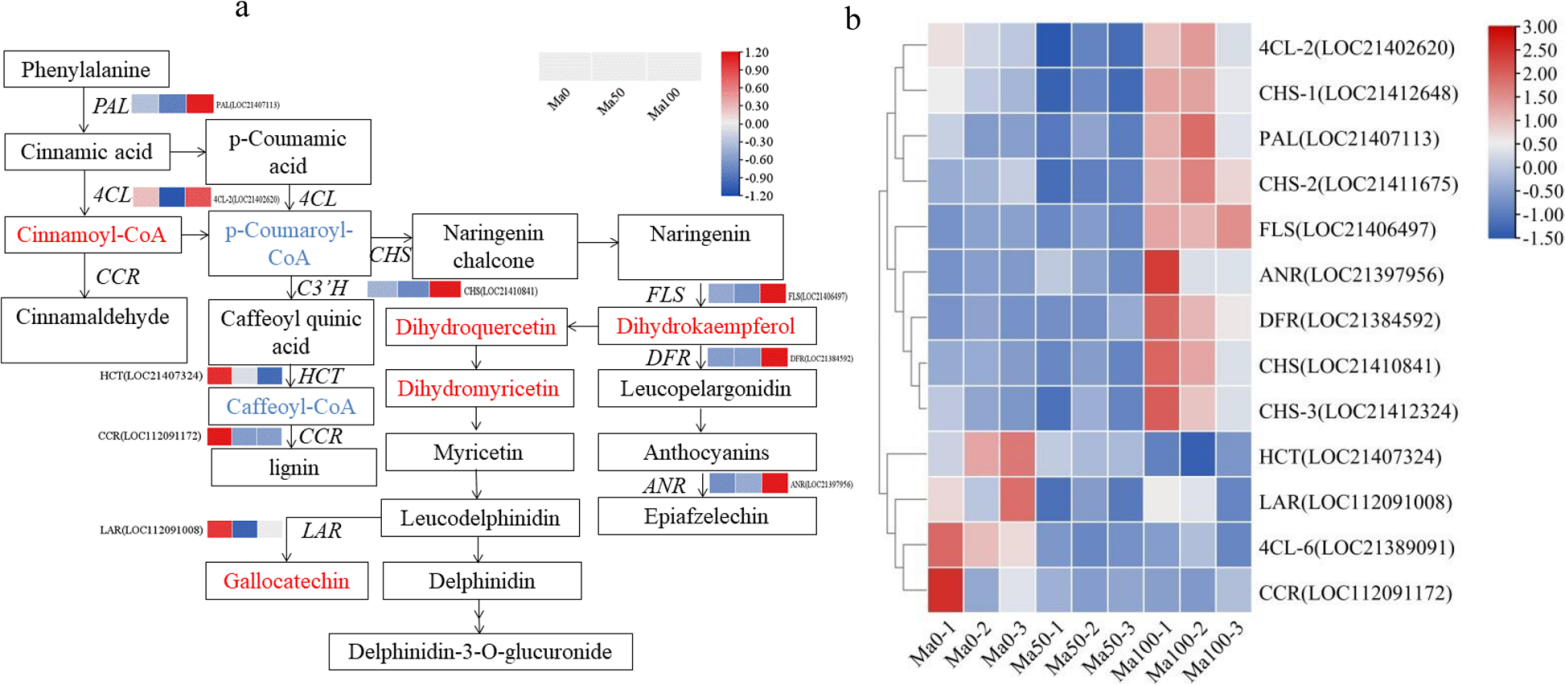
Transcript profiling of genes in the phenylpropanoid and flavonoid biosynthetic pathways. **a** Flavonoid biosynthetic pathway. The pathways were constructed based on the KEGG pathway database and the literature [40]. Each box represents the difference in the expression of related genes under different salt treatments. Red represents a high level of expression, and blue represents a low level. Red and blue indicate upregulated and downregulated metabolites, respectively. *PAL*, phenylalanine ammonia-lyase; *4CL*, 4-coumarate-CoA ligase; *C3’H*, 5-O-(4-coumaroyl)-D-quinate 3’-monooxygenase; *HCT*, shikimate O-hydroxycinnamoyltransferase; *CCR*, cinnamoyl-CoA reductase; *CHS*, chalcone synthase; *FLS*, flavonol synthase; *DFR*, bifunctional dihydroflavonol 4-reductase/flavanone 4-reductase; *ANR*, anthocyanidin reductase; *LAR*, leucoanthocyanidin reductase. **b** Heatmap representation of flavonoid-related gene expression patterns. The gene expression data are shown as a heatmap depicting log2 (FPKM) values

To predict the molecular mechanism of flavonoid accumulation in mulberry seedlings under salt treatment, we reconstructed the flavonoid biosynthetic pathway (Fig. 12a). Gene function annotation and KEGG enrichment analysis revealed that 140 DEGs were significantly enriched in mulberry flavonoid biosynthesis. The 140 DEGs included 90 involved in flavonoid biosynthesis pathways (such as phenylpropanoid biosynthesis), two *HCT* genes and three *COMT* genes, and 50 DEGs involved in the regulation of anthocyanin or flavonoid biosynthesis, five *PAL* genes, two *4CL* genes, 21 *CHS* genes, one *CCR* gene, four *FLS* genes, three *DFR* genes, two *LAR* genes, and four *ANR* genes (Table S5). The flavonoid biosynthesis pathway starts with the production of 4-coumaroyl-CoA from phenylalanine via *PAL* and *4CL* activities, and the main precursors for 4-coumaroyl-CoA biosynthesis are 4-coumarinyl-CoA and malonyl-CoA, which produce chalcone via *CHS* [40]. As shown in Fig. 12b, the expression of the *PAL* (LOC21407113) gene was significantly upregulated in Ma100 compared with Ma50 (P < 0.01), and the gene was expressed at high levels in Ma100. *4CL* is involved in the accumulation of cinnamoyl-CoA, and the expression of cinnamoyl-CoA reductase-like SNL6 (LOC21403409 and LOC21405777) was significantly upregulated in Ma100 compared with Ma50 (Table S5). Furthermore, the expression of *4CL* (LOC21402620) was significantly upregulated in Ma100 compared with Ma50 (P < 0.01), and the gene was expressed at high levels in Ma100 under salt treatment. Phenolics are generated by *HCT* and *CCR*, and the downregulation of these genes was detected mainly in Ma50. However, the remaining portion enters the flavonoid synthesis pathway to produce chalcone under the action of *CHS*. The expression of *FLS* (LOC21406497), which is involved in the production of dihydrokaempferol, dihydroquercetin and dihydromyricetin, was significantly upregulated in Ma100 vs. Ma0 and Ma100 vs. Ma50 (P < 0.01), and the expression levels of dihydrokaempferol and dihydroquercetin were elevated in Ma100 and decreased in Ma50 and Ma0; dihydromyricetin was elevated in Ma100 and Ma50 and decreased in Ma0 (Fig. 8). *DFR* and *ANR* are key enzymes involved in anthocyanin synthesis in plants. Under salt treatment, the expression of *DFR* (LOC21384592) and *ANR* (LOC21397956) was significantly upregulated in Ma100 vs. Ma0 and in Ma100 vs. Ma50, and these genes accumulated at a high level in Ma100. *LAR*, involved in the production of gallocatechin, was expressed at a reduced level in Ma100 and Ma50 and at a high level in Ma0 (Fig. 12b), and the expression level of gallocatechin-(4α → 8)-gallocatechin was elevated in Ma100 and decreased in Ma50 and Ma0 (Fig. 8). These results indicated that *PAL*, *4CL*, *CHS*, *FLS*, *DFR* and *ANR* were the key genes involved in flavonoid accumulation during mulberry seed germination under salt stress.

### Analysis of transcription factors (TFs) involved in flavonoid biosynthesis

Salt treatment activated transcription factors involved in flavonoid biosynthesis in mulberry seedlings. In the present study, the most numerous TF families (in the top 8 classes) were identified under salt stress; these included AP2/ERF-ERF, MYB, WRKY, bHLH, NAC, C2H2, GRAS, and LOB. We screened TFs associated with flavonoid biosynthesis, including one MYB, two bHLH, and two NAC TFs, under control and salt treatment conditions (Table 1). MYB123 (LOC21395818) and bHLH42 (LOC21407775) were significantly upregulated in Ma100 compared with Ma0 and were positively correlated with the levels of phloretin-2'-O-glucoside (phlorizin) (mws2118) and apigenin; 4',5,7-trihydroxyflavone (ZBN0062), respectively. The levels of NAC (LOC21396473 and LOC21399320) were positively correlated with the levels of catechin (MWSHY0065) in both Ma50 vs. Ma0 and Ma100 vs. Ma0, which indicates that MYB123 (LOC21395818), bHLH42 (LOC21407775) and NAC (LOC21396473 and LOC21399320) may positively regulate flavonoid biosynthesis. In contrast, the level of bHLH 6 (LOC21406208) was negatively correlated with that of quercetin-3-O-glucoside (isoquercitrin) (mws0091), which may negatively regulate the accumulation of flavonoids in mulberry plants.

**Table 1:** Transcription factors involved in flavonoid biosynthesis

TFs	Gene name	Compounds	metaID	Ma50 vs. Ma0			Ma100 vs. Ma0		
				Log2FC	PCC	*p* value	Log2FC	PCC	*p* value
MYB123	LOC21395818	Phloretin-2'-O-glucoside (Phlorizin)	mws2118	0.289	0.848	0.004	1.409	0.817	0.004
bHLH42	LOC21407775	Apigenin; 4',5,7-Trihydroxyflavone	ZBN0062	0.160	0.933	0.000	1.630	0.806	0.009
bHLH6	LOC21406208	Quercetin-3-O-glucoside (Isoquercitrin)	mws0091	0.870	-0.825	0.006	1.017	-0.825	0.006
NAC	LOC21396473	Catechin	MWSHY0065	0.570	0.809	0.008	0.570	0.809	0.008
NAC	LOC21399320	Catechin	MWSHY0065	0.570	0.816	0.007	0.570	0.817	0.007

PCC refers to Pearson's correlation coefficient, when PCC is positive, it means that there is a positive correlation between the two samples and when PCC is negative, it means that there is a negative correlation between the two samples

### Validation of genes and key enzymes (proteins) involved in flavonoid biosynthesis via qRT‒PCR and PRM

**Fig. 13:**
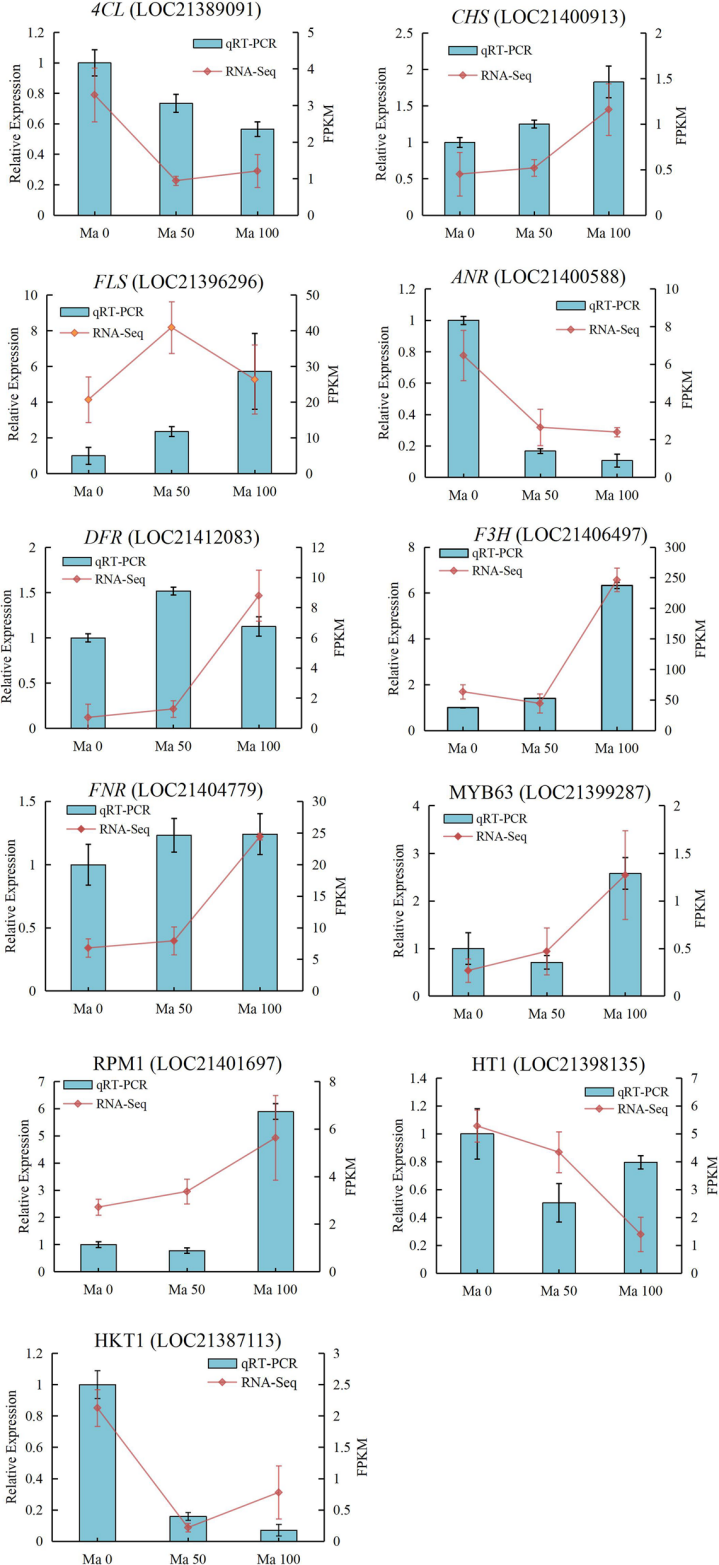
qRT‒PCR validation of 11 genes in mulberry seedlings under different salt treatment concentrations. The FPKM values, which represent the relative expression levels of DEGs, are shown as red lines, corresponding to the Y-axis on the right side. qRT‒PCR validation was performed using a bar chart corresponding to the Y-axis on the left side

To assess the accuracy of the transcriptomic profiles obtained by RNA sequencing, 11 DEGs were selected for qRT‒PCR validation. Among these 11 DEGs, 7 DEGs were involved in the flavonoid biosynthetic pathway and included *4CL*, *CHS*, *FLS*, *ANR*, *DFR*, *F3H* and *FNR*. *CHS*, *FLS*, *DFR*, *F3H* and *FNR* were highly expressed in Ma50 and Ma100 compared to Ma0, while *4CL* and *ANR* were expressed at low levels. Furthermore, MYB63 and RPM1 (a disease resistance protein) were more highly expressed in Ma100 than in Ma0 and Ma50. HT1 (serine/threonine-protein kinase) and HKT1 (high-affinity K^+^ transporter) were expressed at low levels in Ma50 and Ma100 compared to Ma0. The results indicated that the qRT‒PCR validation results were largely consistent with the FPKM values (Fig. 13).

To validate the flavonoid metabolomics data, we randomly selected 6 key enzymes (proteins) involved in flavonoid biosynthesis for quantitative detection via the PRM, including chalcone synthase, 4-coumarate-CoA ligase, phenylalanine ammonia-lyase, flavonol synthase, dihydroflavonol 4-reductase and cinnamoyl-CoA reductase. In Ma50/Ma0, the expression levels of 4-coumarate-CoA ligase and cinnamoyl-CoA reductase were significantly reduced according to both the flavonoid metabolomics and the PRM analysis results. The expression levels of chalcone synthase, flavonol synthase and dihydroflavonol 4-reductase in Ma100/Ma0 significantly increased according to both the flavonoid content and the PRM analysis results. Overall, the PRM results demonstrated that the relative expression levels of the selected key enzymes (proteins) were consistent with the accumulation of flavonoids (Table 2).

**Table 2:** Quantitative validation of key enzymes (proteins) involved in flavonoid biosynthesis by PRM analysis

Gene ID	Protein Name	Protein Symbol	Fold Change		PRM	
			Ma50/Ma0	Ma100/Ma0	Ma50/Ma0	Ma100/Ma0
LOC21410841	chalcone synthase	CHS	0.697	2.482	0.731	3.282
LOC21402620	4-coumarate-CoA ligase	4CL-2	0.478	1.213	0.467	1.404
LOC21407113	phenylalanine ammonia-lyase	PAL	0.810	1.558	0.954	1.742
LOC21406497	flavonol synthase	FLS	0.699	3.887	0.813	2.770
LOC21384592	dihydroflavonol 4-reductase	DFR	1.000	11.865	1.018	8.657
LOC112091172	cinnamoyl-CoA reductase	CCR	0.310	0.318	0.291	0.563

## Discussion

Mulberry is considered an economically important crop in sericulture. Salt stress is a major abiotic stress factor affecting crop production, and understanding the mechanisms underlying the seed germination response to salt stress can help to improve crop tolerance [41]. Studies on alfalfa have shown that the growth of cotyledons, roots and hypocotyls significantly decreases with increasing salt concentration, resulting in decreased seedling fresh weight [42]. In the present study, the length of the roots of mulberry plants decreased significantly with increasing salt concentration, while the decrease in fresh weight was not significant. In addition, seed germination is one of the most important growth and developmental processes in plants; it is extremely sensitive to salt stress and is subjected to the coordinated interactions of multiple genes and metabolites [43]. Mulberry plants are rich in flavonoids and other secondary metabolites, which play a wide range of roles in defense against biotic and abiotic stresses [18]. Combined transcriptomic and metabolomic studies can provide us with new, large-scale information about shifted flavonoids and potential modifications in gene expression networks [44]. In the present study, the involvement of flavonoids in regulating the tolerance of mulberry seedlings to salt stress was investigated via 15 days of mulberry seed germination. Comprehensive transcriptomic and metabolomic analyses were used to explore the effects of salt stress on the accumulation of flavonoids in mulberry and the underlying gene expression mechanism, and the results provide a theoretical basis for the study of the regulatory effects of salt stress on seed germination and flavonoid biosynthesis in mulberry.

### Key DFMs during mulberry seed germination under salt stress

Flavonoids are major secondary metabolites that are widely distributed and play important roles in mulberry plants [45]. In recent years, studies on mulberry flavonoids have focused on their concentration, species differences and pharmacological effects [46]. In contrast to the limitations of previous studies in determining the types of flavonoids in mulberry, the results of this study greatly enrich our understanding of the types and composition of flavonoids in mulberry. In this paper, 145 DFMs were screened into 9 groups, 40 flavonols, 32 flavones, 28 other flavonoids, 16 chalcones, 14 flavanones, 8 flavanonols, 3 flavanols, 3 proanthocyanidins, and 1 tannin, which is similar to the results of a previous study on the biosynthesis of mulberry leaf flavonoids [46]. The present study showed that the levels of 89 DFMs, including quercetin-3-O-glucoside (isoquercitrin), kaempferol (3,5,7,4'-tetrahydroxyflavone), quercetin-7-O-glucoside, taxifolin (dihydroquercetin) and apigenin (4',5,7-trihydroxyflavone), may indicate that the accumulation of flavonoid compounds helps mulberry plants defend themselves against salt stress. Studies have shown that quercetin and its glycosides have potent antioxidant effects and play important roles in reducing oxidative stress and increasing salinity tolerance in *A. thaliana* [47]. Quercetin-3-O-glucoside has been shown to have high antioxidant activity, and moderate salinity induces the synthesis of quercetin-3-O-glucoside [48]. In addition, maize plants exhibited a significant increase in relevant metabolites, including quercetin 3-O-glucoside, kaempferol 3-O glucoside, and quercetin 3-methyl ether, under salt stress [49], which is similar to the results of this study. Taxifolin (dihydroquercetin) and its derivatives are flavonoids with important medicinal value that are widely distributed in plants and play important roles in the defense against biotic and abiotic stresses [50, 51]. Apigenin (4,5,7-trihydroxyflavone) is an abundant flavone aglycone widely distributed in many herbs, fruits and vegetables that has been shown to inhibit lipid peroxidation, eliminate free radicals and enhance endogenous antioxidant defense mechanisms [52, 53]. Furthermore, it has been shown that apigenin promotes flavonoid accumulation in rice under NaCl stress conditions, suggesting that the flavonoid apigenin can improve salt tolerance in rice [52]. Therefore, in this study, we hypothesized that these enriched flavonoids are key metabolites involved in mulberry defense against salt stress.

### Changes in transcriptomic and metabolomic levels during mulberry seed germination under salt stress

Gene expression and metabolite levels are closely related during mulberry seed germination under salt stress. Transcriptomic analyses revealed that the metabolic processes significantly enriched in DEGs in the three comparison groups Ma50 vs. Ma0, Ma100 vs. Ma0 and Ma100 vs. Ma50 were involved in flavonoid biosynthesis, phenylpropanoid biosynthesis and biosynthesis of secondary metabolites, respectively. Metabolomic analyses revealed that the pathways in which DFMs were enriched in the three comparison groups Ma50 vs. Ma0, Ma100 vs. Ma0 and Ma100 vs. Ma50 included flavonoid biosynthesis, biosynthesis of secondary metabolites and metabolic pathways. KEGG coenrichment analysis showed that DEGs and DFMs were significantly enriched in metabolic pathways, biosynthesis of secondary metabolites and flavonoid biosynthesis during mulberry seed germination. Furthermore, flavonoids and phenylpropanoids usually play important roles in plant stress tolerance, and both of these biosynthesis pathways are important for seed germination [54, 55]. 12 genes in the flavonoid biosynthesis (ko00941) pathway were associated with 6 metabolites with differential abundance between Ma50 and Ma0, where upregulated dihydromyricetin (mws0744) was negatively correlated with *ANR* (LOC21400588), *FLS* (LOC21406496) (Fig. 11a). Sixty-seven genes were associated with 11 metabolites whose abundance differed between Ma100 and Ma0, among which upregulated kaempferol (3,5,7,4'-tetrahydroxyflavone) (mws1068) was positively correlated with *CHS* (LOC112091300), *DFR* (LOC21384592); upregulated dihydrokaempferol (mws1094) and *DFR* ( LOC21404779), *FLS* (LOC21406497) and *FLS* (LOC21406498) were positively correlated; upregulated dihydroquercetin (mws0044) was negatively correlated with *ANR* (LOC21400588), *FLS* (LOC21406496); upregulated dihydromyricetin (mws0744) and *ANR* (LOC21400588), *FLS* (LOC21406496) were negatively correlated (Fig. 11b). Fifty genes were strongly associated with 4 metabolites whose abundance differed between Ma100 and Ma50, and upregulated kaempferol (3,5,7,4'-tetrahydroxyflavone) (mws1068) was positively associated with 27 genes including *CHS* (LOC112091300), *DFR* (LOC21384592) and *FLS* (LOC21406497) (Fig. 11c). The results indicated that kaempferol, dihydrokaempferol, dihydroquercetin and dihydromyricetin may be involved as specific metabolites in the transcriptional regulation of key genes. Studies have indicated that these flavonoids have antioxidant capacity, which can scavenge excessive reactive oxygen species and mitigate the damage of salt stress on plant cell membranes and enzyme systems [56, 57]. In addition, these flavonoids can regulate the physiological and metabolic processes of plants and enhance the salt tolerance of plants [58, 59].

### Key DEGs encoding involved in flavonoid biosynthesis during mulberry seed germination under salt stress

Flavonoids have positive effects on environmental stress and multiple functions [32]. In the present study, seed germination under salt stress was affected by the transcript levels of key genes involved in the flavonoid biosynthesis pathway. The flavonoid biosynthesis pathway begins with the phenylalanine metabolic pathway, and *PAL* is the first enzyme in the phenylpropionate pathway that catalyzes the nonoxidative deamination of phenylalanine to trans-cinnamic acid. In the present study, the expression of the *PAL* gene (LOC21407113) was significantly upregulated in Ma100 compared with Ma50, and this gene was expressed at a high level in Ma100, which may have been induced by salt stress. Valifard et al. [60] showed that 100 mM NaCl induced *PAL* expression (12–18 times) in the leaves of two medicinal *Salvia* plants (*Salvia mirzayanii* and *Salvia macrosiphon*), which increased *PAL* activity. The *4CL* gene encodes a key enzyme involved in the biosynthesis of lignin and flavonoids and plays an important role in plant stress [61, 62]. Two *4CLs* (*4CL-2* and *4CL-6*) were identified in our study: *4CL-2* expression was significantly upregulated in Ma100 vs. Ma50, *4CL-6* expression was significantly downregulated in Ma50 vs. Ma0 and in Ma100 vs. Ma0, and *4CL-2* expression was greater than that of *4CL-6* (Table S5), suggesting that salt stress in mulberry seeds may affect *4CL* expression to regulate germination. *CHS* is the first key enzyme in the conserved flavonoid synthesis pathway [63]. It has been reported that overexpression of the *CHS* gene in okra of *A. thaliana* under salt stress increases the flavonoid content, thus conferring resistance against salt stress-related damage [64]. In this paper, we identified 21 *CHS* genes that were upregulated in Ma100 compared with Ma50 (Table S5), and upregulated kaempferol was positively correlated with *CHS* (LOC112091300) under salt stress, which may indicate that *CHS* is a key gene involved in the accumulation of flavonoid end products during mulberry seed germination and plays a critical role in protecting mulberry plants from salt stress damage.

*FLS* not only is a key structural gene involved in the regulation of flavonoids but can also be induced by salt stress [65], thus synergistically improving plant salt tolerance. Zhou et al. [66] reported that overexpression of the tea (*C. sinensis*) *FLS* gene in tobacco (*Nicotiana tabacum* L.) resulted in a significant increase in the levels of flavonols and their derivatives and in the total flavonoids in tobacco. In the present study, four *FLS* genes were identified, and the expression of the *FLS* gene (LOC21406497) was significantly upregulated in Ma100 vs. Ma0 and in Ma100 vs. Ma50, and upregulated dihydrokaempferol was positively correlated with *FLS*(LOC21406497) and *FLS*(LOC21406498). This may indicate that overexpression of the *FLS* gene increased the content of dihydrokaempferol, thereby improving the salt tolerance of mulberry. In addition, the expression of genes associated with flavonoids, such as *PAL*, *CHS*, and *FLS*, increased under salt stress, which enhanced plant resistance [67]. *DFR* and *ANR* are key enzymes involved in anthocyanin synthesis in plants. The *DFR* gene regulates anthocyanin accumulation and has been shown to improve salt tolerance in *Brassica napus* [68].. In this paper, three *DFR* genes and four *ANR* genes were identified. The expression of *DFR* (LOC21384592) and *ANR* (LOC21397956) was significantly upregulated in Ma100 vs. Ma0 and in Ma100 vs. Ma50, the transcripts accumulated at high levels in Ma100, and upregulated dihydrokaempferol and *DFR* (LOC21404779) were positively correlated, which may indicate that overexpression of the *DFR* gene leads to the accumulation of dihydrokaempferol to resist salt stress. In addition, *4CL*, *DFR* and *ANR* are key branch point genes that regulate flavonoid accumulation [69]. In summary, we hypothesized that *PAL*, *4CL*, *CHS*, *FLS*, *DFR* and *ANR* are key genes involved in flavonoid accumulation during mulberry seed germination under salt stress. The qRT‒PCR validation results showed that the expression trends of most genes were strongly correlated with the RNA-seq results (Fig. 13, *P* < 0.05), and the results of the PRM identification of key enzymes involved in flavonoid synthesis proved the reliability of the flavonoid content assay results. These results showed that the flavonoid biosynthesis pathway is important for mulberry seed germination under salt stress. Our results are consistent with previous studies reporting that flavones and flavonols accumulate in plants exposed to salt stress and that they enhance plant salinity tolerance by scavenging ROS [49, 70].

### Transcription factors involved in flavonoid biosynthesis during the germination of mulberry plants under salt stress

The flavonoid biosynthesis pathway involves a complex transcriptional regulatory network consisting of multiple transcription factors, and transcription factors such as those of the MYB, bHLH, and NAC families have been shown to play important roles in regulating flavonoid synthesis in plants [71, 72]. MYB transcription factors can regulate the expression of genes encoding enzymes related to flavonoid synthesis and thus efficiently regulate the biosynthesis of flavonoids [71]. AtMYB11, AtMYB12 and AtMYB111 in *A. thaliana* independently activate the expression of flavonol synthesis-related genes, such as *CHS*, *CHI* and *FLS* [73]. Overexpression of FtMYB1 and FtMYB2 in tobacco led to increased accumulation of proanthocyanidins by affecting the expression of *PAL*, *F3H*, *FLS*, *DFR*, and *ANR*, and FtMYB15 induced the expression of genes in the early and late stages of flavonoid biosynthesis [74, 75]. bHLH transcription factors are among the largest families of transcription factors and play important roles in the regulation of a wide range of secondary metabolites, including flavonoid compounds. Li and Zachgo [76] reported that the bHLH transcription factor TCP3 can interact with AtMYB12 and AtMYB111 to promote the bioaccumulative synthesis of flavonols. bHLH subfamily III members promote the biosynthesis of flavonoids in apple (*Malus pumila*) and strawberry (*Fragaria* × *ananassa*) plants and enhance the plant defense system [77]. In strawberry, coexpression of FvMYB10 with FvbHLH33 significantly activated the promoters of the anthocyanin biosynthesis structural genes *FvDFR* and *FvUFGT*, whereas knockdown of FvMYB10 or FVbHLH33 significantly reduced the activity of the *FvDFR* promoter [78]. Plant growth and development, such as seed germination, secondary wall formation and lateral root development, are regulated by NAC transcription factors [79]. NAC transcription factors are modulated by environmental stimuli, phytohormones and miRNAs and play important regulatory roles in plants subjected to abiotic stresses such as salinity, drought and cold [80]. Under salt stress, NAC13-overexpressing transgenic poplar plants exhibited enhanced salt tolerance, whereas NAC13-suppressed plants were more sensitive to salt stress [81]. Overexpression of transcription factors such as those of the MYB, bHLH, and NAC families can stimulate the expression of corresponding downstream genes, which in turn improve plant tolerance to saline and alkaline stresses [82], as shown for the soybean variety GmMYB173 [27], grape variety VvbHLH1 [83], and apple variety MdNAC52 [84], which regulate the expression of structural genes or regulatory genes involved in flavonoid biosynthesis in plants, thus leading to the accumulation of flavonoids. This study showed that MYB123 (LOC21395818), bHLH42 (LOC21407775), and NAC (LOC21396473 and LOC21399320) may positively regulate flavonoid biosynthesis, suggesting that the MYB, bHLH, and NAC transcription factors are the major classes of transcription factors regulating the biosynthesis of flavonoids in mulberry plants under salt stress. It has been speculated that flavone biosynthesis in mulberry resources may be regulated by transcription factors such as the R2R3-MYB, bHLH and bZIP transcription factors [85].

## Conclusions

In this study, we investigated the regulatory mechanism of mulberry seed germination under salt stress via comprehensive transcriptomic and metabolomic analysis. The flavonoid metabolomic results showed that salt stress resulted in increased levels of 89 DFMs, including quercetin-3-O-glucoside (isoquercitrin), kaempferol (3,5,7,4'-tetrahydroxyflavone), quercetin-7-O-glucoside, taxifolin (dihydroquercetin) and apigenin (4',5,7-trihydroxyflavone). Transcriptomic analysis revealed that most of the DEGs were enriched in flavonoid biosynthesis (ko00941), phenylpropanoid biosynthesis (ko00940) and biosynthesis of secondary metabolites (ko01110). Combined analysis of the flavonoid metabolomic and transcriptomic data indicated that *PAL*, *4CL*, *CHS*, *FLS*, *DFR*, and *ANR* were the key genes involved in flavonoid accumulation during mulberry seed germination under salt stress. Members of 3 transcription factor families (MYB, bHLH and NAC) were also found to be involved in the regulation of flavonoid accumulation. The qRT‒PCR validation results showed that the expression levels of 11 DEGs, including 7 genes involved in flavonoid biosynthesis, under different salt treatment concentrations were consistent with the transcriptomic data, and the results of PRM identification of key enzymes for flavonoid synthesis proved the reliability of the flavonoid content assay results. This study provides a new perspective for elucidating the mechanism of flavonoid biosynthesis in the regulation of mulberry seed germination under salt stress. Future research will focus on identifying the genes involved and linking flavonoids with various plant hormones to understand how flavonoids participate in the salt tolerance of mulberry plants.

### Supplementary Information

**Supplementary material 1.**

**Supplementary material 2.** 

## Data Availability

The datasets analysed during the current study are available in the National Center for Biotechnology Information repository, [https://www.ncbi.nlm.nih.gov/bioproject/PRJNA1063633, accession number- PRJNA1063633].
