# Supplementary figures and images for: Integrated transcriptomic and metabolomic analyses elucidate the mechanism of flavonoid biosynthesis in the regulation of mulberry seed germination under salt stress
Source: BMC Plant Biol. 2024 Feb 21;24:132. doi: 10.1186/s12870-024-04804-3 (PMC10880279; doi:10.1186/s12870-024-04804-3)

a

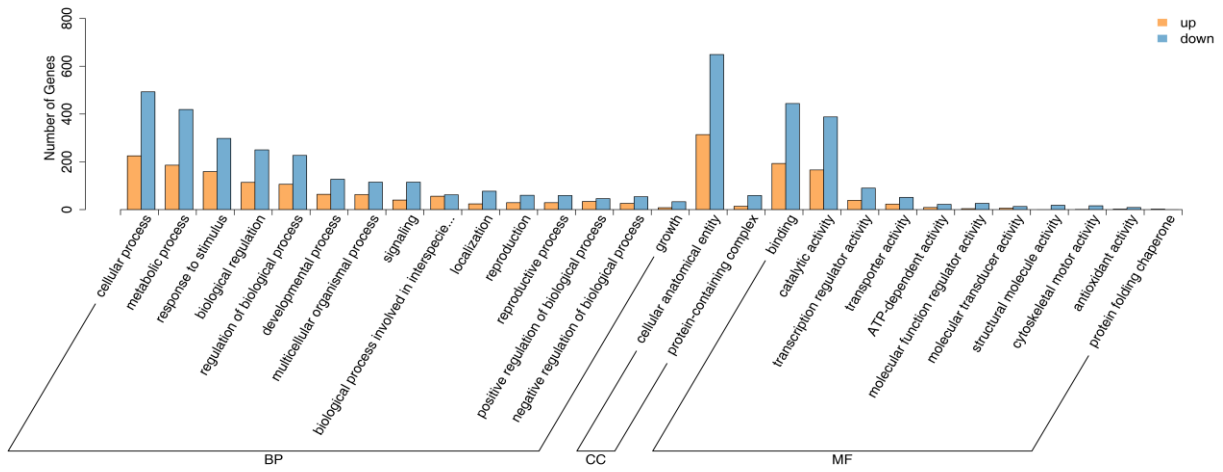

b

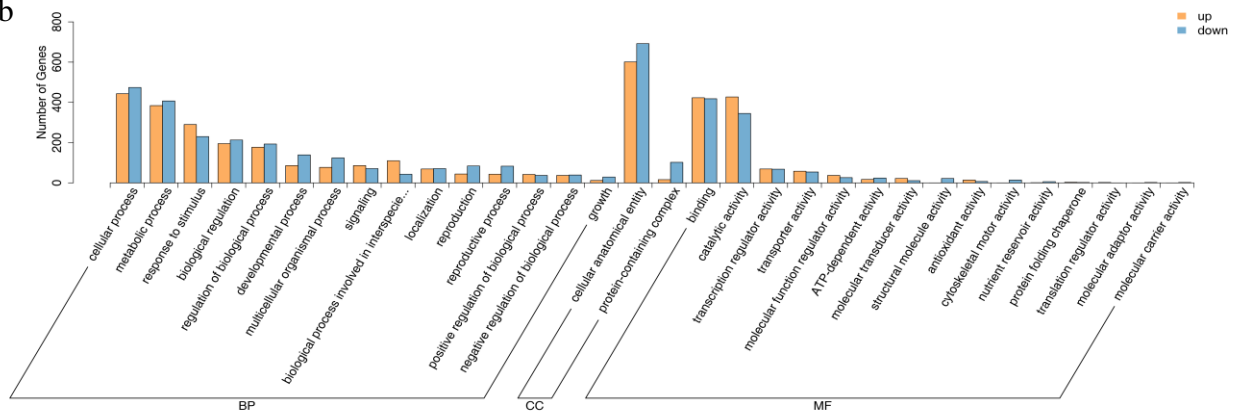

c

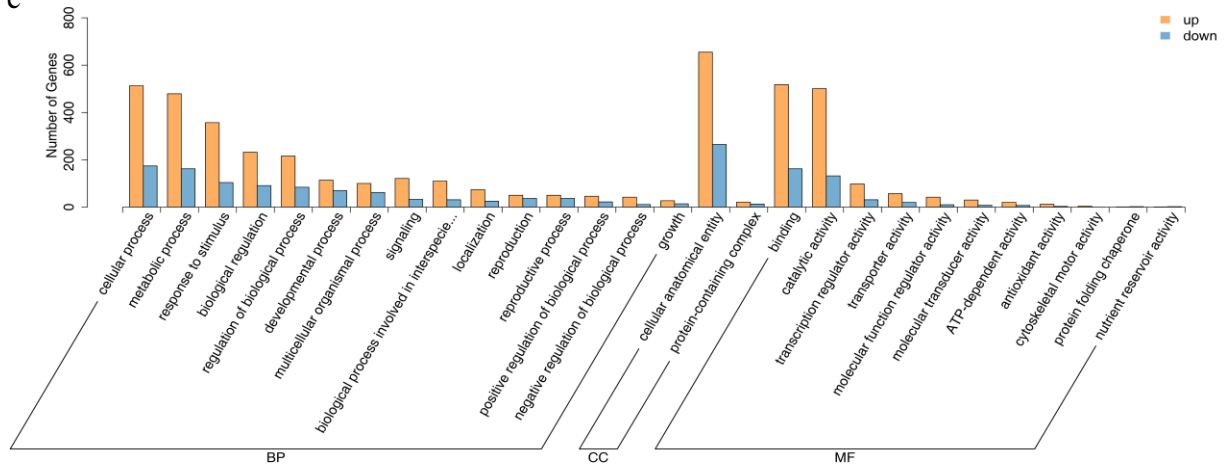

Supplement: Supplementary file 2 — Supplementary material 2. [file 12870_2024_4804_MOESM2_ESM.zip › Supplementary Figure/Figure S1. GO classification of DEGs in the three comparison groups.pdf]
